# Phytochemical profile, physicochemical, antioxidant and antimicrobial properties of *Juniperus phoenicea* and *Tetraclinis articulate*: *in vitro* and *in silico* approaches

**DOI:** 10.3389/fchem.2024.1397961

**Published:** 2024-09-13

**Authors:** Ayoub Asbabou, Touijer Hanane, Aman Allah Gourich, Farhan Siddique, Aziz Drioiche, Firdaous Remok, Soukaina Saidi, Imad Adadi, Hamid Khamar, Khalid S. Almaary, Amare Bitew Mekonnen, Mohammed Bourhia, Amale Bouzoubaa, Touriya Zair

**Affiliations:** ^1^ Research Team of Chemistry of Bioactive Molecules and the Environment, Laboratory of Innovative Materials and Biotechnology of Natural Resources, Faculty of Sciences, Moulay Ismaïl University, Meknes, Morocco; ^2^ School of Pharmaceutical Science and Technology, Tianjin University, Tianjin, China; ^3^ Department of Botany and Plant Ecology, Scientific Institute, University Mohammed V in Rabat, Rabat, Morocco; ^4^ Department of Botany and Microbiology, College of Science, King Saud University, Riyadh, Saudi Arabia; ^5^ Department of Biology, Bahir Dar University, Bahir Dar, Ethiopia; ^6^ Laboratory of Biotechnology and Natural ResourcesValorization, Faculty of Sciences, Ibn Zohr University, Agadir, Morocco

**Keywords:** *Juniperus phoenicea*, *Tetraclinis articulata*, essential oils, antioxidant activity, antimicrobial activity

## Abstract

**Introduction:**

This research aims to explore the molecular composition, antioxidant capabilities, and antibacterial effects of the essential oils from *Tetraclinis articulateata* and *Juniperus phoenicea*.

**Methods:**

Essential oils were extracted using hydrodistillation. Gas chromatography combined with mass spectrometry was used to determine the chemical makeup of essential oils. Two methods are used to assess the antioxidant activity of essential oils: the reduction of iron (ferric reducing antioxidant power or frap) and the trapping of the free radical 2,2-Diphenyl-1-Picrylhydrazyl (DPPH). The antimicrobial potential of essential oils was assessed using the diffusion method on a solid-state disk in comparison to nine bacterial and seven fungal souches.

**Results and Discussion:**

The essential oil yields from *Tetraclinis articulata* and *Juniperus phoenicea* are 0.46% ± 0.02% and 0.83% ± 0.05%, respectively. According to CG/SM’s chromatographic analyses, the predominant constituent in the essential oil of J. Phoenicea is α-pinène (59.51%), while the main constituents in the essential oil of *T. Articulata*? are Bornyle acetate (18.91%) and camphor (28.48%). The assessment of antioxidant activities reveals intriguing antioxidant qualities in the essential oils of the species under investigation. *T. Articulata* essential oils yield the greatest results in the DPPH and FRAP tests, with CI50 values of around 266.9 ± 5.4 μg/mL and EC50 values of 433.16 ± 4.13 μg/mL, respectively. Except for Staphylococcus epidermidis, Staphylococcus aureus BLACT, and Pseudomonas aeroginosa, the two essential oils have demonstrated significant bactericidal activity against all bacterial and fungal souches (MIC <2 mg/mL et MBC <3.5 mg/mL). The inhibiting effect of these oils on bacterial and fungal development raises potential application areas in the food, cosmetic, and pharmaceutical industries. In addition, the current study investigated the potential antifungal, antibacterial, and antioxidant activities of the essential oils from *Juniperus phoenicea* and *Tetraclinis articulate* plants via the Glide molecular docking methodology, and most of these constituents were observed to be potent therapeutic agents.

## 1 Introduction

Essential oils derived from aromatic and therapeutic plants have been known to exhibit numerous important biological actions since antiquity (Ait-Sidi-Brahim et al., 2019; Bouyahya et al., 2017; Harmouzi et al., 2016). These goods, which are utilized in the food, cosmetics, and pharmaceutical sectors, have a high added value. Numerous clinical research conducted in the past 10 years have demonstrated the antibacterial and antioxidant properties of several essential oils (Atailia and Djahoudi, 2015; Amarti et al., 2008; Nasir Shah et al., 2023). The temperature, altitude, type, and pH of the soil, time of harvest, and methods used for drying and extraction all affect the chemical composition of essential oils. Several studies have demonstrated a connection between the chemical composition of essential oils, particularly the characteristics of their main volatile constituents, and the biological activities of these oils (Satrani et al., 2007). Morocco, due to its geographical location, constitutes a completely original natural setting offering a complete range of Mediterranean bioclimates favoring a rich and varied flora with very marked endemism. The rich vegetation of Morocco is underappreciated in terms of its chemical, sensory, or biological potential. In this context, our work focused on two Moroccan species: *Juniperus phoenicea* and *Tetraclinis articulata*. Both plants belong to the Cupressaceae family known for their Mediterranean essences and characterized by multiple uses in traditional medicine.

The Cupressaceae family is a large and important family of the plant world, classified in the subphylum Gymnosperms, order Coniferales (Adams, 2000). It is the largest family of conifers in terms of genera, and the third in terms of species (Farjon, A. 2010). It brings together twenty-nine genera and more than 130 species (Farjon, 2005; Adams et al., 2009). The genus Juniperus occupies an important place in the Cupressaceae family, with about 60 species distinguished by their hardiness and dynamism (Adams, 1998). The red or Phoenician juniper (*Juniperus phoenicea*) is a bushy shrub or monoecious tree, standing 1–8 m high (Benabid, A. 2000). It is a variable species, characterized by great morphological and biochemical differentiation, which has made it possible to distinguish three subspecies: *J. phoenicea sub spphoenicea, J. phoenicea sub sp*eu-mediterranea and *J. phoenicea var. turbinata* (Adams et al., 2002). *Juniperus phoenicea* is a species whose distribution area is circum-Mediterranean. It is present in North Africa, South Africa, southern Europe, and Asia (Adams, 1998). Red juniper is considered an important medicinal plant, widely used in traditional medicine in many countries. The branches, leaves, and fruits of *J. phoenicea* are used in traditional medicine and their chemical compounds are incorporated into pharmaceutical preparations of particularly antiseptic use attributed to the presence of essential oils (Mansouri et al., 2011). The mixture of leaves and female cones is widely used in traditional Moroccan and Algerian medicine to treat diabetes (Amel, B 2013). The plant is also used for its antihypertensive, anti-inflammatory, antiparasitic, and antiseptic properties (Boudjelal et al., 2013). Previous studies that studied the chemical composition of the essential oils of *J. phoenicea* in different regions on either side of the basin Mediterranean have shown that the major constituent in its oils is α-pinene which has antibacterial, antifungal, and antiseptic properties (Amalich et al., 2015; El-Sawi et al., 2007; Adams et al., 1996). The Berberian or Maghreb thuja (*Tetraclinis articulata*), is a monoecious, long-lived tree (around 500 years), 6–20 m high (Fennane, 1957). The distribution of this species is limited to the southern western Mediterranean region, present in Morocco, Algeria, Tunisia, and Spain (Abbas et al., 2006; Bourkhiss et al., 2015). Berberie thuja is a plant widely used in traditional medicine in several countries, particularly in Morocco. In traditional medicine, the different organs of thuja, notably the leaves and branches, are used in the treatment of intestinal and respiratory infections (Bellakhdar et al., 1991). The essential oils of *T. articulata* stand out for their strong antifungal activity; their spectrum of action is very broad. Consequently, they act on a large number of phytopathogenic fungi (Ghnaya et al., 2016). Several works have been carried out on the chemical composition of the essential oils of *Tetraclinis articulata* showing its richness in oxygenated monoterpenes such as α-pinene, limonene, isobornyl acetate, thymol (El et al., 2010; Harmouzi et al., 2016; Chikhoune et al., 2013; Bourkhiss et al., 2015). Research carried out in the scientific literature indicates that there are few reports of studies on the antioxidant and antimicrobial properties of the essential oils of *J. phoenicea* and *T. articulata* (Saber et al., 2023; Sahib et al., 2022).

*J. phoenicea* and *T. articulata*, have been valued for their healing properties for centuries such as essential oils (Fahim et al., 2017; Lawal and Ogunwande, 2013). Recent scientific investigations have started to uncover the complex phytochemical profiles of these plants, exposing a plethora of bioactive compounds. The physicochemical characteristics, along with strong antioxidant and antimicrobial properties, highlight their potential in pharmacological and nutraceutical applications (Djahafi et al., 2021; Harmouzi et al., 2016; Tahar et al., 2023). This current study examines the extensive phytochemical composition, physicochemical attributes, and antioxidant and antimicrobial efficacy of *J. phoenicea* and *T. articulata*, Middle Atlas Morocco plants emphasizing their potential to improve human health and addressing microbial resistance.

## 2 Materials and methods

### 2.1 Chemicals

Ethyl alcohol, hydrochloric acid, glacial acetic acid and methanol were obtained from Merck (Darmstadt, Germany). Na_2_CO_3_, NaH_2_PO_4_. 2H_2_O, Na_2_HPO_4_, K_3_Fe(CN)_6_, 2,2-diphenyl-1-picrylhydrazyl (DPPH) and KOH were obtained from Fluka (Buchs, Switzerland). Trichloroacetic acid (TCA), Sodium hydroxide and AlCl_3_.6H_2_O were from Riedel-de Haen (Seelze, Germany). Ascorbic acid was from Panreac (Barcelona, Spain). Deionized double distilled water was used for all assays.

### 2.2 Plant material

**FIGURE 1:**
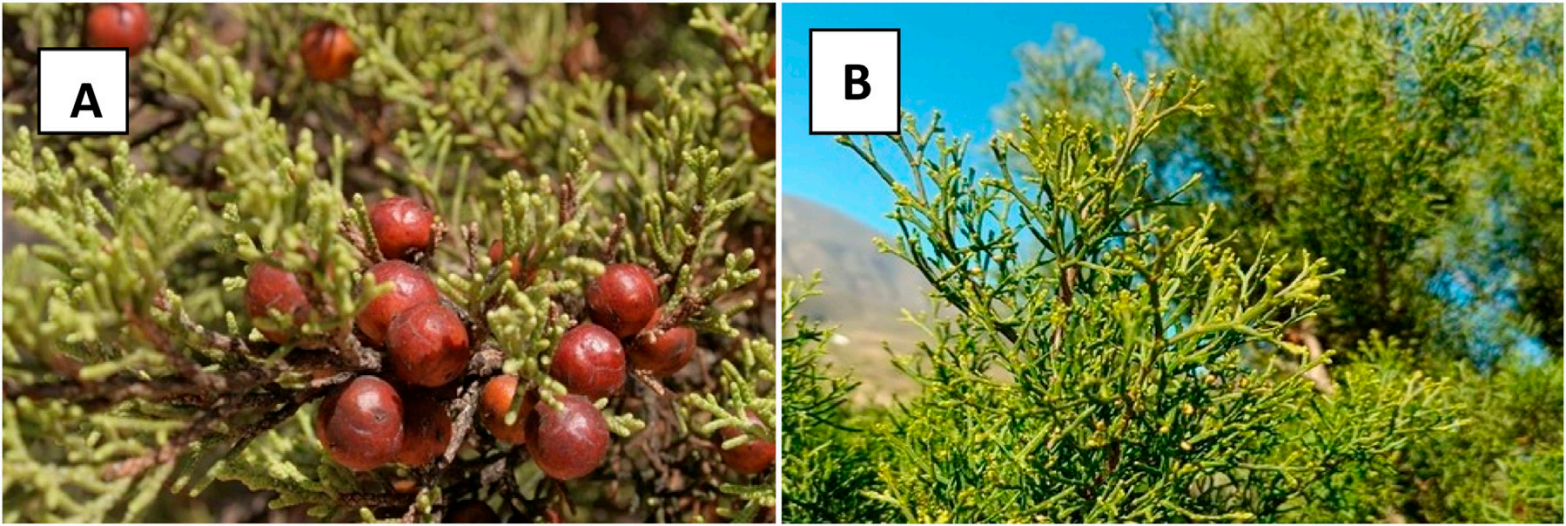
The morphological appearance of the plants studied, **(A)** *J. phoenicea*, **(B)** *T. articulate*.

The leafy branches of *J. phoenicea* were collected in the Boulmane region in March 2022, while those of T. articulata were collected in Rebat Elkheir in June 2022. The two plants were dried for several days at protected from humidity and light. The geographical parameters of the harvesting stations and the morphological appearance of the plants studied are represented in Tables 1, 2; Figure 1. The identification of the plants studied was carried out at the Department of Botany and Plant Ecology, Rabat Scientific Institute.

**TABLE 1:** Harvest sites of the plants studied.

Botanical name	Harvest site		Longitude (y)	Latitude (x)	Altitude (m)
	Region	Locality			
*T. articulata*	Sefrou	Ribate El Kheir	33°48′42″ N	4°24′57″ W	1,250
*J.phoenicea*	Boulmane	Gigo	33°39′02″ N	4°82′86″ W	1,509

**TABLE 2:** The taxonomic classification of the plants studied.

Kingdom	Plantae	Plantae
Class	Equisetopsida	Equisetopsida
Order	Cupressales	Cupressales
Family	Cupressaceae	Cupressaceae
Genus	Juniperus	Tetraclinis
Species	*Juniperus phoenicea* L	*Tetraclinis articulata* (Vahl) Masters

### 2.3 Bacterial and fungal material

Sixteen microbial strains were chosen for their pathogenicity and their frequent involvement in the contamination of foodstuffs: nine bacterial strains [*Staphylococcus epidermidis*, *Staphylococcus aureus* BLACT, *Streptococcus agalactiae* (B), wild *Escherichia coli*, *Escherichia coli* ESBL, *Enterobacter cloacae*, *Klebsiella pneumoniae*, *Proteus mirabilis* and *Pseudomonas aeruginosa*] and seven fungal strains (*Candida albicans*, *Candida dubliniensis*, *Saccharomyces cerevisiae*, *Aspergillus niger*, *Candida tropicalis*, *Candida krusei*, *Candida parapsilosis*). These selected microorganisms are pathogenic, known for their high resistance, invasive and toxic power in humans. They are frequently encountered in many infections in Morocco that pose a clinical and therapeutic problem. These strains were isolated from the hospital environment: Mohamed V-Meknes Provincial Hospital.

### 2.4 Quality control of plant material

#### 2.4.1 Moisture content (MC)

A quantity of 5 g of the fresh plant material was dried in a Memmert-type oven at 105°C for 24 h (to a constant weight). The mass of the dry plant was determined using a precise balance and the water content is given by the Equation 1 below:

$$\text{MC}\%=\frac{\left(m0-m1\right)}{m0}\times 100$$ (1)

m_0_ (g): Initial mass of the plant.
m_1_ (g): Mass after drying.
The result was expressed as a percentage of dry matter.

#### 2.4.2 Determination of pH

The principle consists of adding 10 mL of hot distilled water to a quantity of 2 g of powdered plant material. After filtering, the mixture is left to cool. A pH meter of the HANNA type was used to measure the pH.

#### 2.4.3 ASH content

A quantity of 1 G of the powdered plant material was taken and placed in a previously tared crucible. The whole is introduced into a muffle furnace at 550°C for 48 h, then weighed again after cooling. The organic matter content is calculated by Formula 2 given below:

$$\text{OM}\left(\%\right)=\left(\frac{W1-W2}{TS}\right)X100$$ (2)

OM%: Organic matter;
W_1_: Weight of sample before calcination;
W_2_: Weight of sample after calcination;
TS: Test sample.
The ash content was calculated by the Equation 3:

Ash%=100−MO% (3)

### 2.5 Extraction and estimation of essential oil yield

The extraction of the essential oil was carried out by hydrodistillation in a Clevenger-type apparatus (Clevenger, 1928). The distillation lasts 3 hours after recovery of the first drop of distillate, three distillations were made by boiling 100 g of the plant. Then the resulting oil was dried by adding anhydrous sodium sulfate (Na2SO4). The essential oil is stored at 4°C in the dark. The HE yields are expressed concerning dry matter (in mL/100 g of dry matter).

### 2.6 Analyse chromatographique

The chromatographic analysis of essential oils was carried out using a THERMO ELECTRON chromatograph (Thermo Fischer Scientific, Waltham, Massachusetts, United States): Trace GC Ultra equipped with a DB-5 (5% phenyl-methyl-siloxane) capillary column (30 m × 0.25 mm, film thickness: 0.25 μm), of a flame ionization detector (FID) powered by a mixture of H2/Air-gas. The carrier gas is nitrogen with a flow rate of 1 mL/min. The device is equipped with a split–splitless PVT (Programmed Vaporization Temperature) injector. The injection mode is split (leakage ratio: 1/50, flow rate: 66 mL/min). The injected volume is 1 μL. The experimental temperature increases from 50°C to 200°C with a gradient of 4°C/min. Mass spectrometry is carried out with a gas chromatograph of the THERMO ELECTRON Trace MS system type (THERMO ELECTRON: Trace GC Ultra; Polaris Q MS). Fragmentation is carried out by electronic impact with an intensity of 70 eV. The capillary column is type DB-5 MS (5% phenyl-methyl-siloxane) (30 m × 0.25 mm, film thickness: 0.25 μm). The column temperature increases from 50°C to 200°C at a rate of 4°C/min. Helium is used as a carrier gas with a flow rate of 1.5 mL/min. The injection is done in split mode (leakage ratio: 1/70, flow rate mL/min). The masses listed fall within a range of 30–500 m/z. The device is connected to a computer system managing a library of NIST 98 mass spectra. The identification of the constituents was carried out based on their Kováts Indices (IK) and on gas chromatography coupled with spectrometry of mass (GC/MS).

### 2.7 EO quality control

The physicochemical properties studied were determined according to the standards of the French Standardization Association (AFNOR) and the International Standardization Organization (ISO).

#### 2.7.1 Density

The density of an essential oil at 20°C is the ratio of the density of this oil to the density of water at the same temperature, using a RADWAG-PS 600/C/2 precision electronic balance (RADWAG, Radom 26–600, Poland).

#### 2.7.2 Acid value

This is the amount of potassium hydroxide milligrams required to balance the free acids in 1 G of essential oil. Potassium hydroxide titrated in an ethanolic solution is used to neutralize free acids. The standard NFV 03–906 was used to determine the acid number (Association Francaise de normalisation 1984).

#### 2.7.3 Miscibility with ethanol

The miscibility test described in standard NF ISO 875 consists of gradually adding (from 0.1 mL in 0.1 mL increments up to 20 mL) an ethanol solution of suitable alcoholic strength to a test portion of oil essential (1 mL), at a temperature of 20°C. We then note the volume of ethanol that caused the cloudiness or opalescence (reaching the critical point of complete miscibility) of the essential oil.

#### 2.7.4 Ester value

The ester index is the number of mg of potassium hydroxide necessary for the neutralization of the acids released by the hydrolysis of the esters contained in 1 g of EO. The hydrolysis of the esters present in the EO is done by heating, under defined conditions, in the presence of ethanol previously titrated with KOH and followed by back-dosing of the excess alkali with a titrated solution of hydrochloric acid.

### 2.8 Antioxidant activity

#### 2.8.1 Evaluation of anti-radical activity by trapping the free radical (2,2-diphenyl-1-picrylhydrazyl: DPPH)

The evaluation of the antioxidant activity of essential oils was carried out using the stable radical 2,2-diphenyl-1-picrylhydrazyl (DPPH) method described by (Parejo et al., 2000). In this test, the purple-coloured DPPH has an absorption maximum at 515 nm, is reduced to a yellow compound, diphenylpicrylhydrazine, whose color intensity is inversely proportional to the reduced capacity of the antioxidants present in the medium (Sanchez-Moreno, 2002). The DPPH solution is obtained by dissolving 2.4 mg of the powder in 100 mL of ethanol. 200 μL of the ethanolic solution of the essential oils tested are added to 2.8 mL of the previous DPPH solution at different concentrations. The antioxidant power of the EO tested was estimated by comparison with a reference antioxidant (Ascorbic acid). All tests were performed with three repetitions for each concentration. After an incubation period of 30 min at laboratory temperature, the absorbance is read at 517 nm. The percentage of inhibition of the DPPH radical is calculated according to the following Equation 4:

$$PI\left(\%\right)=\frac{A0-A1}{A0}X100$$ (4)

A_0_ and A_1_ are the absorbance values of the blank and the test sample, respectively.

The antioxidant power is characterized by the IC_50_ parameter.

IC_50_ is inversely related to the antioxidant capacity of a compound, as it expresses the amount of antioxidants required to decrease the free radical concentration by 50%. The lower the IC_50_ value, the greater the antioxidant activity of a compound.

#### 2.8.2 Evaluation of antioxidant activity by the iron reduction method (ferric ion reducing antioxidant power: FRAP)

The reducing power is determined according to the method recommended by (Karagözler et al., 2008). One ml of different concentrations of each essential oil from 0.5 to 3.0 mg/mL diluted in ethanol is mixed with 2.5 mL of 0.2 M phosphate buffer solution at pH 6.6 and 2.5 mL potassium ferricyanide [K3Fe(CN)6] at 1%. The mixtures are incubated at 50°C for 20 min and then cooled. Then, 2.5 mL of trichloroacetic acid (10%) is added. 2.5 mL of the supernatant of each concentration is mixed with 2.5 mL of distilled water and 0.5 mL of 0.1% iron chloride (FeCl_3_). Ascorbic acid is used as the reference antioxidant in this experiment. The absorbances are read against a blank at 700 nm using an R-200 closed-type spectrophotometer (Thermo Fischer Scientific, Waltham, Massachusetts, United States). The results make it possible to calculate the effective concentration (EC_50_), the concentration of the extract corresponding to an absorbance equal to 0.5, obtained by interpreting the linear regression curve (density of the optics as a function of the different concentrations).

#### 2.8.3 Antimicrobial activity of essential oils of *J. phoenicea* and *T. articulata*

To evaluate the antimicrobial activity of essential oils we used the aromatogram method. The diffusion of the antimicrobial agent in the inoculated medium results from a gradient of the antimicrobial. When the concentration of the antimicrobial becomes much diluted, it can no longer inhibit the growth of the bacteria tested; the zone of inhibition is demarcated. The diameter of this zone of inhibition correlates with the minimum inhibitory concentration (MIC) for the particular bacteria/antimicrobial combination, the zone of inhibition corresponds inversely to the MIC of the assay. Generally, the smaller the zone of inhibition, the lower the concentration of antimicrobial needed to inhibit the growth of microorganisms.

### 2.9 Glide molecular docking methodology

The glide molecular docking method is an effective tool for evaluating various interactions of the ligands with the target proteins (Zheng et al., 2013). It has been incorporated to visualize the antifungal, antibacterial and antioxidant activities of investigated compounds under study (Azeem et al., 2023; Luo et al., 2018; Ali et al., 2023).

#### 2.9.1 Selection of target proteins

The crystal structures of the antifungal, antibacterial, and antioxidant proteins of interest (PDB ID: 5EQB), (PDB ID: 4Q9M), and (PDB ID: 1K4Q) were retrieved from the RCSB protein data bank (https://www.rcsb.org/) (Berman et al., 2000).

#### 2.9.2 Pre-processing of target protein structures

The Protein Preparation Wizard module of the Schrodinger Maestro platform v12.8 (Yusuf et al., 2023) was employed to prepare the protein structures. The module assigned jobs to prepare bond orders, add hydrogen atoms and establish zero-order bonds to metals, as well as create di-sulfate bonds followed by changing the seleno-methionines into methionine, and then filling up the missing side chains. The structures were finally optimized by energy minimization followed by hydrogen bond optimization under the OPLS4 force field (Lu et al., 2021).

#### 2.9.3 Pre-processing of ligand structures

The LigPrep module of the Schrodinger Maestro platform was employed to prepare the co-crystallized ligands of targeted proteins and the essential oils isolated from the *Juniperus phoenicea* and *Tetraclinis articulate*.

#### 2.9.4 Glide docking

Glide docking of the target proteins and ligand molecules was executed by the Schrodinger Maestro platform’s Glide docking module (Friesner et al., 2006). The SP pose viewer analyzed the docked ligand and protein interactions and then generated the optimal pose. The ligand interaction module generated a 2D interaction diagram of the ligand-protein complex which was then visualized to investigate the interaction between the ligand molecules and the selected target proteins during the binding process through the resulting SP posture.

## 3 Results and discussion

### 3.1 Quality control of plant material

The collected samples underwent quality control at the laboratory level by measuring several characteristic parameters such as moisture content, pH, and Ash. The results are grouped in Table 3.

**TABLE 3:** Quality control results of plant material.

Plant	Humidity level (%)	pH	Ash (%)
*Juniperusphoenicea*	13.19	6.29	13.38
*Tetraclinisarticulata*	16.11	5.98	11.44

#### 3.1.1 Humidity level

The samples studied contain water; *J. phoenicea* and *T. articulata* give moisture contents of around 13.19% and 16.11% respectively (Table 3). Before drying, the plant has a humidity level of 70%–90%. The objective of drying is to obtain a product stabilized in the air and without risk of degradation or contamination. To do this, the humidity level must be lowered to around 12% (Thibaut Joliet, 2015). This means that the samples dried under laboratory conditions are of good quality.

#### 3.1.2 pH

The plants studied have a weakly acidic character (pH < 7). It should be noted that pH plays a determining role during chemical and biochemical reactions and can influence the stabilizing properties of an essential oil (antioxidant and antimicrobial effects). Consequently, this result can lead to a good stabilizing character against microorganisms (Ouis et al., 2015).

#### 3.1.3 Ashes

The percentage of total ash provides information on mineral content because minerals are not transformed into volatile substances at high temperatures, unlike organic matter. The total ash contents observed on *J. phoenicea* and *T. articulata* are of the order of 13.38% and 11.44% respectively (Table 3). It should be noted that the ash content varies depending on the species studied, the part of the plant used and the place of harvest.

### 3.2 Chemical yield and composition of essential oils

The yields of essential oils obtained from the samples of *J. phoenicea* and *T. articulata* are respectively 0.83% ± 0.05% and 0.46% ± 0.02% (Table 4).

**TABLE 4:** Yields and organoleptic properties of essential oils.

	Yields (%)	Color	Smell
*J. phoenicea*	0.83 ± 0.05	Pale yellow	Strong and characteristic of junipers
*T.articulata*	0.46 ± 0.02	Pale yellow	Strong and Balsamic

The essential oils of *J. phoenicea* and *T. articulata* exhibit characteristic organoleptic properties. They are colorless to pale yellow liquids with a viscous appearance and an odor reminiscent of the plant. The essential oil yield of *J. phoenicea* is lower than those reported by; Mansouri et al. (2011) (0.90%) and Amalich et al. (2015) (1.71%) in the Midelt region in Morocco. On the other hand, the yield obtained is higher than those of the red junipers of Greece (0.21% for the branches), and of the turbinata subspecies of Spain (0.30% for the branches) (Adams et al. 1996).

**FIGURE 2:**
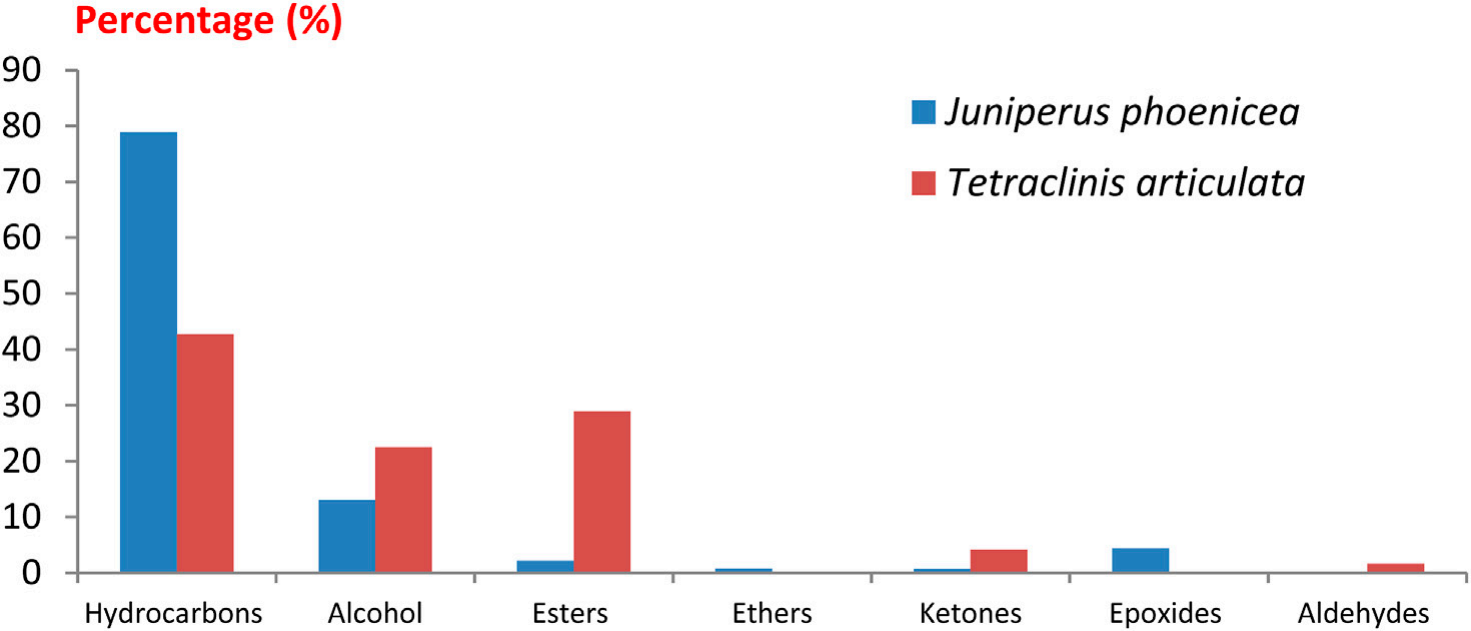
Distribution of chemical families identified in the essential oils of *Juniperus phoenicea* and *Tetraclinis articulate*.

**FIGURE 3:**
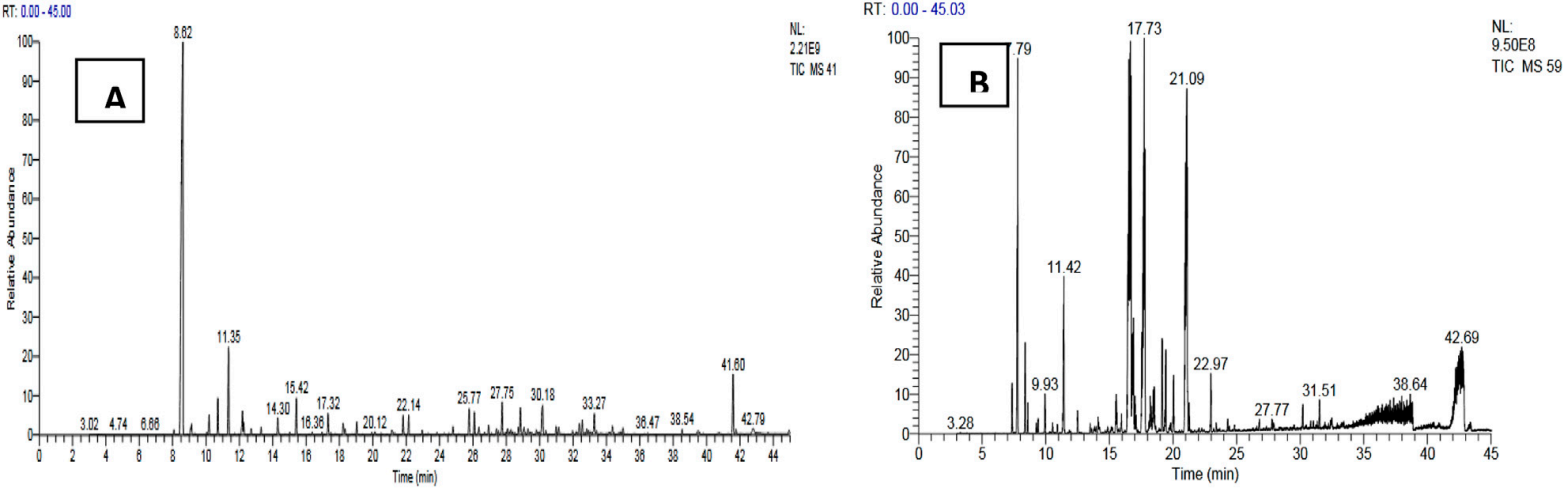
Chromatographic profiles of the studied essential oils, **(A)**: EO of *Juniperus phoenicea*, **(B)** EO of *Tetraclinis articulate*).

A comparison of our results with literature data shows that the yield of essential oils from *T. articulata* is slightly higher than those reported by Zerkani et al. (2019) and Bourkhiss et al. (2015) which obtained a yield of the order of 0.41%. However, this yield is lower than those obtained by Harmouzi et al. (2016) (0.57% ± 0.05%) and Sadiki et al. (2018) (0.84% ± 0.01%). The difference in essential oil yield can be attributed to several factors, including the organ used, species origin, harvest time, and drying time (Moldão-Martins et al., 2002). The results obtained show that both essential oils are dominated mainly by hydrocarbons followed by alcohol, esters and ketones with varying proportions. We note the absence of ethers and epoxides in the essential oil of *T. articulata* and the absence of aldehydes in the essential oil of *J. phoenicea* (Figure 2). The GC/MS analyses of the EO of the plants studied have allowed to make the chromatographic profiles illustrated in Figure 3.

Analysis of the chemical composition of *J. phoenicea* essential oil made it possible to identify 35 compounds which represent a total of 99.95% (Table 5). The essence of *J. phoenicea* consists mainly of α-pinene (59.51%), accompanied by other compounds at relatively low levels: Caryophyllene (3.42%), germacrene D (3.05%), Caryophyllene oxide (2.97%) and Cubenol (2.55%). This oil is characterized by its richness in hydrocarbon monoterpenes (63.94%). Numerous studies have revealed that the essential oils of Phoenician juniper, native to the north of the Mediterranean basin, are dominated by α-pinene including Morocco (Amalich et al., 2015), Algeria (Bekhechi et al., 2012), Egypt (El-Sawi et al., 2007), this confirms our results.

**TABLE 5:** Chemical composition of *J. phoenicea* essential oil.

Compound	IK	Chemical formula	Percentage (%)
α-pinene	939	C_10_H_16_	59.51
Camphene	954	C_10_H_16_	0.64
β-pinene	979	C_10_H_16_	1.22
Myrcene	990	C_10_H_16_	1.27
p-Cymene	1,024	C_10_H_14_	0.60
Limonene	1,029	C_10_H_16_	0.70
Linalool	1,100	C_10_H_18_O	1.22
Pinocarveol	1,139	C_10_H_16_O	0.46
β-Elemene	1,390	C_15_H_24_	0.68
Caryophyllène	1,408	C_15_H_24_	3.42
γ- Elemene	1,436	C_15_H_24_	1.00
Humulene	1,454	C_15_H_24_	1.53
Germacrene D	1,481	C_15_H_24_	3.05
Muurola-4 (14),5-diene	1,493	C_15_H_24_	0.91
epi-Cubebol	1,494	C_15_H_26_O	0.71
α-Muurolene	1,500	C_15_H_24_	0.63
γ- amorphene	1,512	C_15_H_24_	0.56
Cubebol	1,515	C_15_H_26_O	1.19
γ- Cadinene	1,523	C_15_H_24_	2.55
Nootkatene	1,518	C_15_H_22_	0.66
Liquloxide	1,536	C_15_H_26_O	0.46
Hedycaryol	1,548	C_15_H_26_O	1.77
Cadineneether	1,558	C_15_H_24_O	0.78
Maaliol	1,567	C_15_H_26_O	1.26
Caryophyllene oxide	1,583	C_15_H_24_O	2.97
Salvial-4 (14)-en-1-one	1,594	C_15_H_24_O	0.70
Citronellyl	1,625	C_15_H_28_O_2_	1.07
Cubenol	1,628	C_15_H_26_O	2.55
α-Muurololepi	1,642	C_15_H_26_O	0.82
β-Eudesmol	1,650	C_15_H_26_O	0.54
Khusinol	1,680	C_15_H_24_O	0.53
Juniperolacetate	1,685	C_17_H_28_O_2_	1.12
Esudesme-4 (15),7-dien-1 β-ol	1,688	C_15_H_24_O	0.90
Shyobunol	1,689	C_15_H_26_O	1.07
Manool oxide	1,987	C_20_H_34_O	0.95
**Hydrogenated monoterpenes%**			**63,94**
**Hydrogenated sesquiterpenes%**			**14,99**
**Oxygenated monoterpenes %**			**21,07**
**Total %**			**99,95**

The profile of the chromatogram obtained during the GC/MS analysis of the essential oils of *Tetraclinis articulate* vealed the presence of 21 compounds of which 70.72% are monoterpene derivatives and 28.93% are monoterpene hydrocarbons (Table 6). It appears that the majority of components of *Tetraclinis articulate* essential oil are monoterpenes. Five compounds seem to be in the majority: Camphor (28.48%), Bornylacetate (18.91%), Borneol (14.83%), Methyllinoleate (9.02%), α-Pinene (7.44%). The rest of the identified compounds have relatively low contents with values between 0.42% for Myrtenol and 3.98% for Limonene. Given these results and comparison with other work, more or less similar results were reported by Bourkhiss et al. (2015) which shows that the main constituents are: bornyl acetate (30.6%), camphor (18.6%) and α-pinene (16.8%). The same results were obtained by Zerkani et al. (2019) whose main compounds are: bornyl acetate (38.54%) and α-pinene (6.71%).

**TABLE 6:** Chemical composition of *Tetraclinis articulate* essential oil.

Compound	IK	Chemical formula	Percentage (%)
Tricyclene	926	C_10_H_16_	0.85
α-Pinene	939	C_10_H_16_O	7.44
Camphene	954	C_10_H_16_O	1.28
β-Pinene	979	C_10_H_16_O	0.67
Limonene	1,029	C_10_H_16_O	3.98
α-Campholenal	1,126	C_10_H_16_O	0.98
Camphor	1,146	C_10_H_16_O	28.48
Trans-Verbenol	1,144	C_10_H_16_O	2.05
Camphene hydrate	1,149	C_10_H_18_O	2.06
Pinocarvone	1,164	C_10_H_14_O	0.47
Borneol	1,169	C_10_H_18_O	14.83
Myrtenal	1,195	C_10_H_14_O	0.68
Terpineol<α->	1,188	C_10_H_18_O	1.03
Myrtenol	1,195	C_10_H_16_O	0.42
Verbenone	1,205	C_10_H_14_O	2.48
Carveol < trans->	1,216	C_10_H_16_O	0.76
Carveol < cis->	1,229	C_10_H_16_O	1.37
Carvone	1,243	C_10_H_14_O	1.23
Bornylacetate	1,285	C_12_H_20_O_2_	18.91
Terpinylacetate<α->	1,349	C_12_H_20_O_2_	1.00
Methyllinoleate	2,085	C_19_H_34_O_2_	9.02
**Hydrogenated monoterpenes %**			**70.97**
**Hydrogenated sesquiterpenes %**			**0**
**Oxygenated monoterpenes**			**28.93**
**Total**			**99.9**

### 3.3 Physico-chemical characteristics of essential oils

The results of the physicochemical characteristics studied are grouped in the following Table 7.

**TABLE 7:** Physico-chemical characteristics of essential oils.

	*Density*	*Acid index*	*Miscibility with ethanol (V/V)*	*Ester index*
*J. phoenicea*	0.863	1.08	3	73.1
*T. articulata*	0.925	1.22	5	44.7

The acid number gives an idea of the level of free acids. In our study, this index is certainly within the norms (Acid index <2), which shows that our essences are well preserved (low quantity of free acids). The higher the ester index, the better the quality of an essential oil. Our oils reveal an ester index in compliance with AFNOR, standards (Ester index >12). We note that the ester index of the essential oils of *J. phoenicea* is higher than that of *T. articulata*. The miscibility with ethanol depends directly on the composition of the EO, in particular its proportions of hydrophilic and lipophilic compounds. The essential oils of *J. phoenicea* and *T. articulata* are miscible with 95% ethanol with ethanol volumes of 3 mL and 5 mL respectively.

### 3.4 Antioxidant activity of essential oils

Two methods were chosen for their ease of implementation and their reliability in the evaluation of the antioxidant activity of the essential oils of *J. phoenicea* and *T. articulata*. These were the 1,1-Diphenyl -2-picrylhydrazyl (DPPH) test and the FRAP test.

#### 3.4.1 DPPH method

**FIGURE 4:**
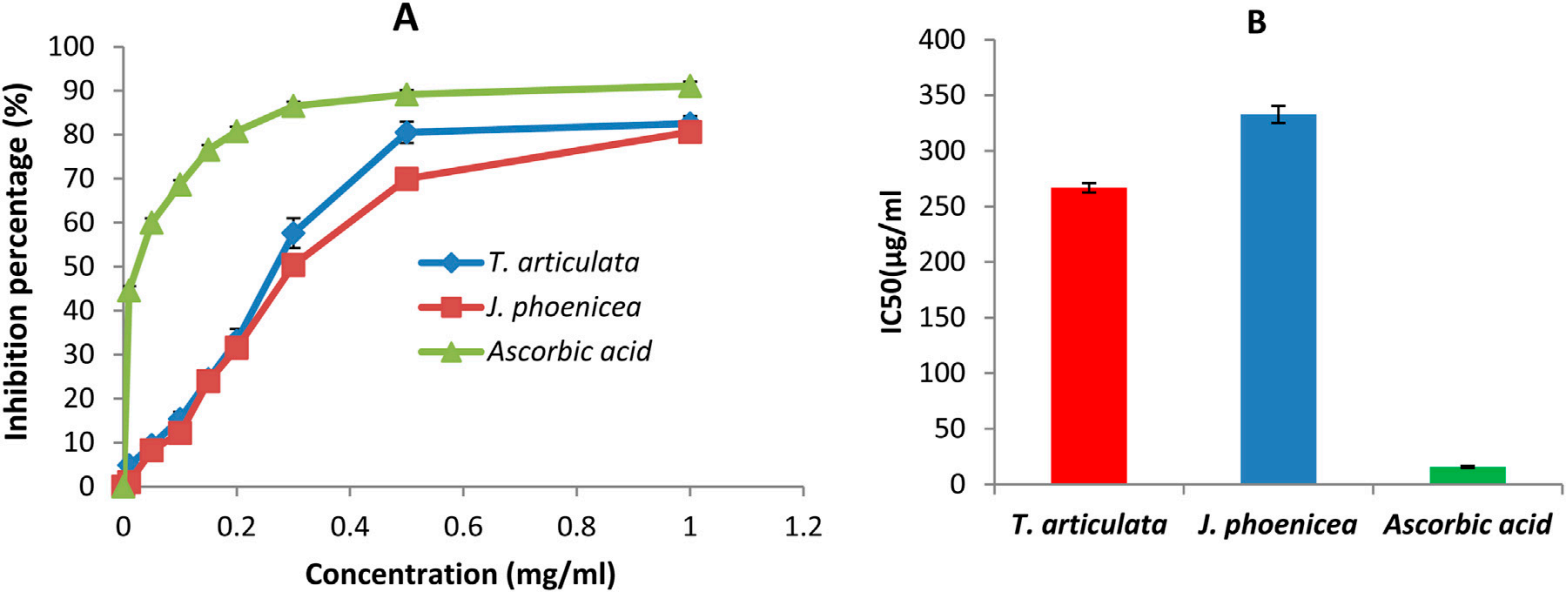
DPPH radical scavenging assay results **(A)**: Percentage inhibition of essential oils and ascorbic acid on DPPH, **(B)** IC_50_ values).

The antioxidant activity of a compound corresponds to its ability to resist oxidation (Rice-Evans et al., 1995). Many methods are currently used to evaluate this activity. The DPPH radical has been widely used to study the anti-radical activity of different plant extracts. The reduction of this radical is accompanied by its transition from the purple color characteristic of the DPPH solution to the yellow color measurable by spectrophotometry at 514–518 nm. The results shown in the curve (Figure 4A) illustrate the percentages of the anti-radical activity of the different oils tested concerning the free radical DPPH. These results show that the essential oils of *J. phoenicea* and *T. articulata* have good anti-radical activity but remain less effective than those of ascorbic acid. At the high concentration of 1 mg/mL, the percentages of DPPH reduction obtained are: 82.50% ± 1.72, 80.58% ± 1.6% and 91.07% ± 0.98 for the essential oils of T. articulata, *J. phoenicea* and ascorbic acid respectively.

The IC_50_ values found for all the essential oils of *J. phoenicea* and *T. articulata* are represented in Figure 4B. By comparing the EC_50_ of the oils tested compared to that of ascorbic acid, we notice that the anti-radical activity of all our oils is lower than the DPPH radical trapping capacity of ascorbic acid (IC_50_ = 15.89 ± 1.4 μg/mL). We also note that the essential oil of *T. articulata* is the most active with an IC_50_ of around 266.9 ± 5.4 μg/mL followed by the essential oil of *J. phoenicea* with a value of 332.8 ± 6.1 μg/mL Table 8. According to the literature, the antioxidant activity of *T. articulata* has been studied by several authors. The majority of this work confirms our results. Indeed, Djouahri et al. (2015) studied the antioxidant activity of the essential oil of thuja leaves from Algeria. The results of the DPPH test showed better activity, with an IC_50_ of around 252.49 ± 6.14 μg/mL, a value comparable to that of our study (266.9 ± 5.4 μg/mL). Note that Camphor (28.48%), Bornyl Acetate (18.91%), Borneol (14.83%), Methyllinoleate (9.02%), α-pinene (7.44%) are the majority constituents of this essential oil. Regarding the results of the antioxidant activity of *J. phoenicea* essential oils, our values are more important in comparison with the results of previous work such as those done by El Jemli et al. (2020) who found an IC_50_ = value 18,780 ± 0.27 μg/mL. It should be noted that *J. phoenicea* essential oils are very rich in α-pinene (Tepe et al., 2005) report that α-pinene is known for its weak antioxidant effect. Therefore, the antioxidant activity of these oils is probably due to the presence of other compounds.

**TABLE 8:** IC_50_ values of essential oils and Ascorbic Acid.

EO	IC_50_(µg/mL)
*T. articulate*	266.9 ± 5.4
*J. phoenicea*	332.8 ± 6.1
*Ascorbic acid*	15.89 ± 1.4

#### 3.4.2 FRAP method

**FIGURE 5:**
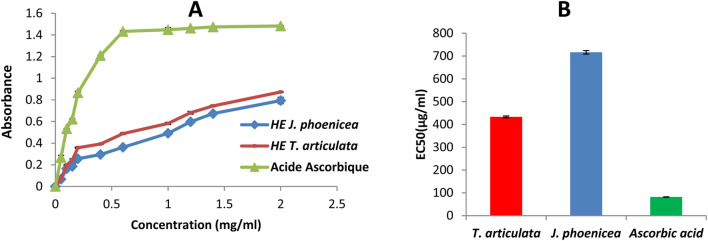
Ferric Reducing power assay results **(A)**: Evaluation of reducing power of essential oils and ascorbic acid, **(B)**: EC_50_ values).

In our work, we tested, using the FRAP method, the ability of essential oils to reduce ferric iron Fe^3+^ to ferrous iron Fe^2+^, and the results obtained allowed us to draw curves for each oil. The results obtained show that the essential oils tested have dose-dependent antioxidant activity (Figure 5A). All our oils have antioxidant activities that are significantly lower than that of the reference (ascorbic acid), for the latter, the reduction is almost complete from a concentration of 0.5 mg/mL (Figure 5A).

The antioxidant capacity of our different extracts is determined from the EC_50_ (Figure 5B). The results obtained show that the capacity of our essential oils to reduce iron is much lower than that of ascorbic acid (EC_50_ = 81.85 ± 0.9 µg/mL). This reduction is much greater in *T. articulata* oils (EC_50_ = 433.16 ± 4.13 μg/mL). However, the essential oils of *J. phoenicea* reveal a low reducing power (EC_50_ = 716.5 ± 7.52 μg/mL) (Table 9).

**TABLE 9:** EC_50_ values.

	EC_50_ (µg/mL)
*T. articulate*	433.16 ± 4.13
*J. phoenicea*	716.5 ± 7.52
*Ascorbicacid*	81.85 ± 0.9

The results obtained show that the essential oils of *J. phoenicea* and *T. articulata* presented low reducing activities compared to those reported by EL JEMLI, et al. (2016) with EC_50_ of the order of 135.68 ± 0.62 and 148.18 ± 0 43 μg/mL respectively. However, the essential oils of *T. articulata* have greater reducing power than those found by Boussaid (2017), EC_50_ = 48.28 ± 0.08 mg/mL.

### 3.5 Antimicrobial activity of essential oils

The determination of the inhibition parameters (MIC and MBC) makes it possible not only to confirm, quantify and compare the activities but also to characterize the nature of the effect revealed by an extract on a given microorganism. The results obtained with the essential oils of *J. phoenica* and *T. articulata* are presented in Table 10.

**TABLE 10:** Determination of the MIC and the MBC of essential oils.

Microorganism		References	MIC/mbc(mg/mL)			
			*J. phoenica*		*T. articulata*	
			MIC	MBC	MIC	MBC
Fungi	Candida *albicans*	C. a	0.6	1.2	2.5	2.5
	*Candida* *dubliniensis*	C. d	1.2	2.5	5	5
	*Saccharomyces cerevisiae*	Sac. C	0.6	0.6	1.2	1.2
	*Aspergillus niger*	Asp. N	0.3	0.3	1.2	1.2
	*Candida tropicalis*	C. t	0.15	0.3	0.3	0.6
	*Candida* *krusei*	C. kr	1.2	1.2	5	5
	*Candida* *parapsilosis*	C. par	0.6	1.2	1.2	2.5
Bacteria	*Staphylococcus* *epidermidis*	5,994	2.5	5	5	5
	*Staphylococcus aureus* BLACT	4IH2510	2.5	2.5	2.5	2.5
	*Streptococcus* *agalactiae*(B)	7DT1887	0.3	0.6	1.2	2.5
	*Escherichia coli* *sauvage*	3DT1938	0.3	0.6	5	5
	*Escherichia coli* BLSE	2DT2057	0.3	0.6	1.2	2.5
	*Enterobacter cloacae*	02EV317	1.2	2.5	5	5
	*Klebsiella pneumoniae*	3DT1823	0.6	1.2	2.5	2.5
	*Proteus mirabilis*	2DS5461	1.2	2.5	1.2	2.5
	*Pseudomonas aeruginosa*	2DT2138	2.5	2.5	>5	>5

MIC, minimum inhibitory concentration; MBC, minimum bactericidal concentration.

The results obtained show that the strongest antibacterial activity was obtained with the essential oil of *J. phoenica* with MIC values that vary between 0.3 and 2.5 mg/mL. All bacterial and fungal strains except *Staphylococcus epidermidis*, *Staphylococcus aureus* BLACT and *Pseudomonas aeroginosa*, are very sensitive to the essence of *J. phoenica* (MIC <2 mg/mL and MBC< 3.5 mg/mL). These results are supported by several studies that have also proven the antimicrobial activity of *J. phoenica* essential oils (Bouzouita et al., 2008; Mansouri et al., 2011; Amalich et al., 2015). The antimicrobial activity of *J. phoenica* essential oils can be explained by its chemical profile rich in terpene hydrocarbons, notably α-pinene. The latter presents several biological activities: antibacterial, anti-inflammatory, antiviral, expectorant, sedative, herbicide and insect repellent (Ghanmi et al., 2007).

The essential oil of *T. articulata* has shown remarkable antimicrobial activity against a range of microorganisms, such as *S. cerevisiae*, *Aspergillus niger*, *Candida tropicalis*, *Candida parapsilosis*, *Streptococcus agalactiae* (B), *Escherichia coli* ESBL, and *Proteus mirabilis*. Nevertheless, its efficacy was restricted when confronted with various fungal and bacterial strains, including *Candida albicans*, *Candida dubliniensis*, *Candida krusei*, *Staphylococcus epidermidis*, *Staphylococcus aureus* BLACT, wild *Escherichia coli*, *Enterobacter cloacae*, *Klebsiella pneumoniae*, and *Pseudomonas aeruginosa*, as their minimum inhibitory concentrations (MICs) surpassed 2 mg/mL. In addition, it was observed that the minimum bactericidal concentrations (MBCs) exceeded 2 mg/mL, suggesting a diminished bactericidal capacity. The results align with previous research (Chikhoune et al., 2013) that indicated limited effectiveness of Thuja essential oils against *Pseudomonas aeruginosa* and *Escherichia coli*, with MICs >5 mg/mL. The antimicrobial activity of *T. articulata* essential oil is a result of its chemical composition, which consists of camphor, bornyl acetate, α-pinene, and borneol. These compounds are well-known for their strong antimicrobial properties, as supported by studies conducted by (Rabib et al., 2020; Angioni et al., 2003).

### 3.6 Glide molecular docking results

Tables 5, 6 show the constituents isolated by GC-MS analysis of *Juniperus phoenicea* and *Tetraclinis articulate* respectively which served as ligands to investigate various therapeutic activities.

Table 11 represents the glide molecular docking data involving the co-crystallized ligand and the hit essential oil constituent (ligands) against the target proteins of interest i.e., antifungal, antibacterial, and anti-oxidant target proteins and reflects the docking results including the essential parameters such as DScore, GScore, Glide Emodel, the polar interactions, the hydrogen bonding with relative distance measured in angstroms (Å), and hydrophobic interactions of the hit compounds (ligands) with the concerned target proteins.

**TABLE 11:** Glide Molecular docking data of interaction of hit compounds and co-crystallized ligand of Juniperus phoenicea and *T. articulate* with the antifungal (5EQB), antibacterial (4Q9M) and anti-oxidant target protein (1K4Q).

Ligands	Docking Score (kcal/mol)	Glide Score (kcal/mol)	Glide emodel (kcal/mol)	H-bonding and Distance in Å	Polar amino acid residues	Hydrophobic interactions
5EQB (antifungal)						
a)5EQB- co-crystallized ligand	−10.217	−12.052	−92.135	HIS468 (2.24) CYS470 (2.09) PHE506 (2.63)	THR130 THR318 SER382 HIS468 THR507 SER508	ALA69, TYR72, LEU95, LEU96, TYR126, LEU129, PHE134, ILE139, TYR140, LEU147, VAL154, PHE236, PRO238, PHE241, LEU307, VAL311, LEU380, LEU383, PHE384, CYS470, ILE471, MET509
b)β-Eudesmol 91,457 (Hit compound of J.phoenica)	−7.270	−7.270	−39.685	SER382 (1.86)	THR130 SER382	TYR126, LEU129, PHE134, TYR140, PHE236, PRO238, PHE241, LEU380, LEU383, PHE384, MET509
B)Bornyl acetate 93,009 (Hit compound of *T. articulate*)	−6.738	−6.738	−34.727	TYR140 (2.04)	THR130 SER382	TYR126, LEU129, PHE134, ILE139, TYR140, PHE236, PHE241, LEU380, LEU383, MET509
4Q9M (Antibacterial)						
e)4Q9M- co-crystallized ligand	−7.597	−7.597	−29.343	ASN30 (1.99) TRP227 (2.74)	SER73 ASN76 ASN146	PHE72, TRP77, VAL84, ILE87, MET88, LEU145
f)γ- Cadinene 92,313 (Hit compound of J.phoenica)	−8.63	−8.63	−39.786	Not found	GLN53	MET27, MET49, LEU52, LEU90, PRO91, TYR95, TYR98, VAL99, LEU102, ILE109, AL125, LEU126, ALA129, LEU141, PHE143, LEU145
F)Carvone 7,439 (Hit compound of *T. articulate*)	−7.310	−7.310	−38.902	Not found	GLN53	MET49, LEU52, PRO91, PHE94, TYR95, TYR98, VAL99, LEU102, ILE109, AL125, LEU126, ALA129, LEU141, PHE143
1K4Q (Antioxidant)						
i)1K4Q-co-crystallized ligand	−7.687	−8.628	−111.177	GLU50 (1.93, 2.01, 2.61) SER51 (2.30) THR162 (2.29) GLY174 (1.97) ASP178 1.82, 1.89	SER51 HIS52 THR57 ASN60 THR156 SER161 THR162 SER172 THR176 GLN182 ASN294	VAL61, ALA155, MET159, PRO160, ILE175
j) γ- amorphene 12,313,019 (Hit compound of J.phoenica)	−5.346	−5.346	−29.566	Not found	THR57 SER177 THR339	CYS58, VAL61, CYS63, PHE181, TYR197, ILE198, MET202, LEU337, LEU338
J)Carveol < cis-> 443,177 (Hit compound of *T. articulate*)	−5.762	−5.762	−32.148	LYS66 (2.03)	THR57 SER177	CYS58, VAL61, CYS63, PHE181, ILE198, MET202, LEU337

**FIGURE 6:**
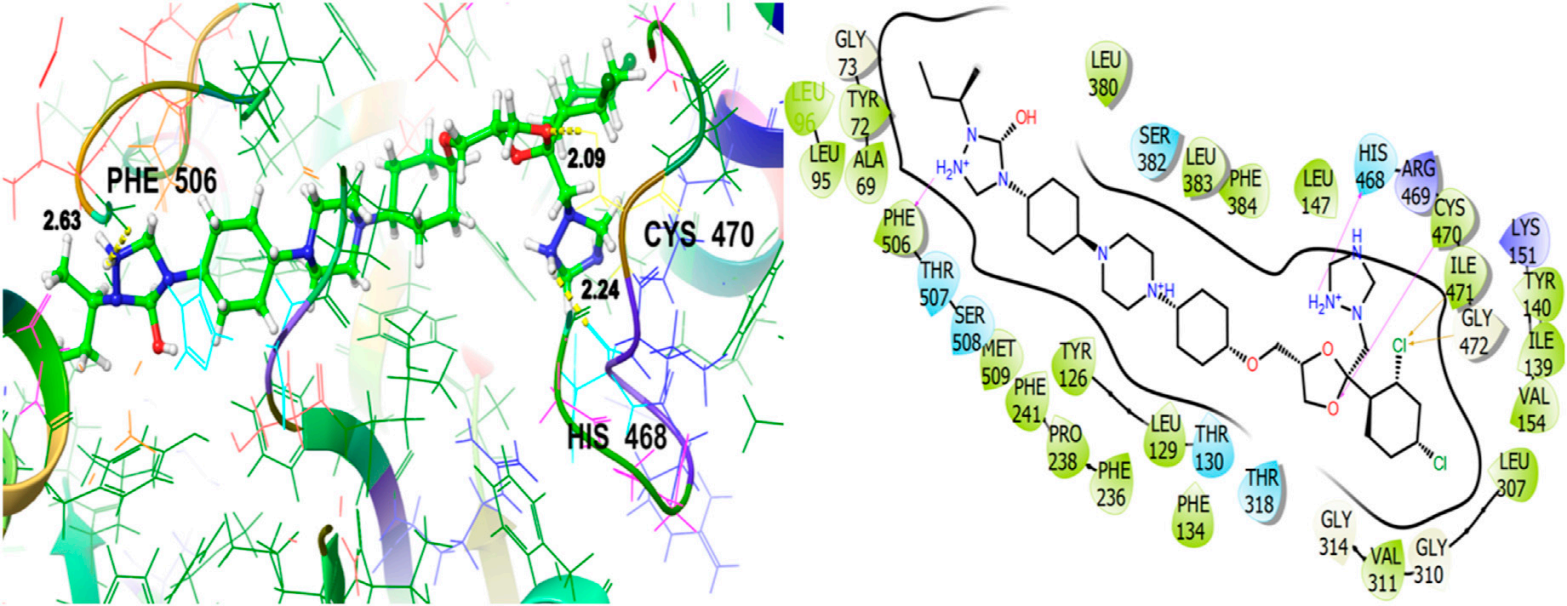
3D and 2D interactive view of co-crystallized ligand with antifungal target protein 5EQB.

**FIGURE 7:**
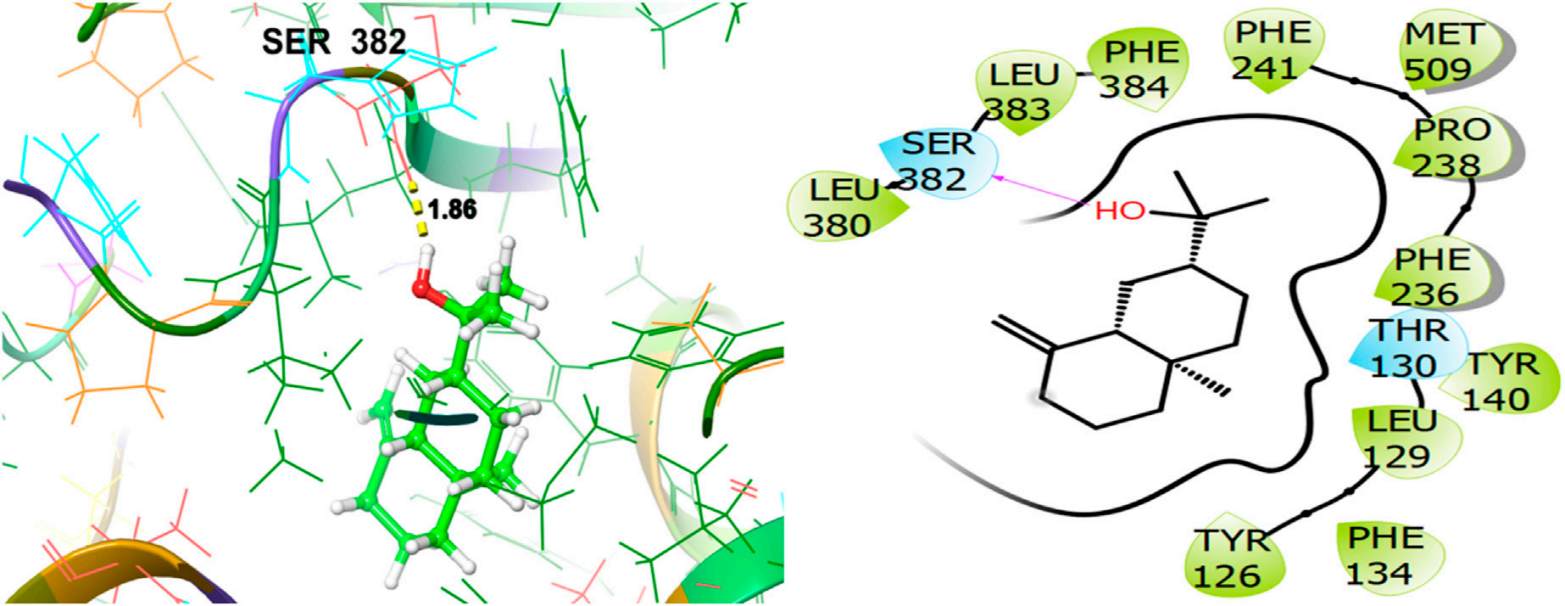
3D and 2D interactive view of β-Eudesmol (isolated from *J. phoenica*) with antifungal target protein 5EQB.

**FIGURE 8:**
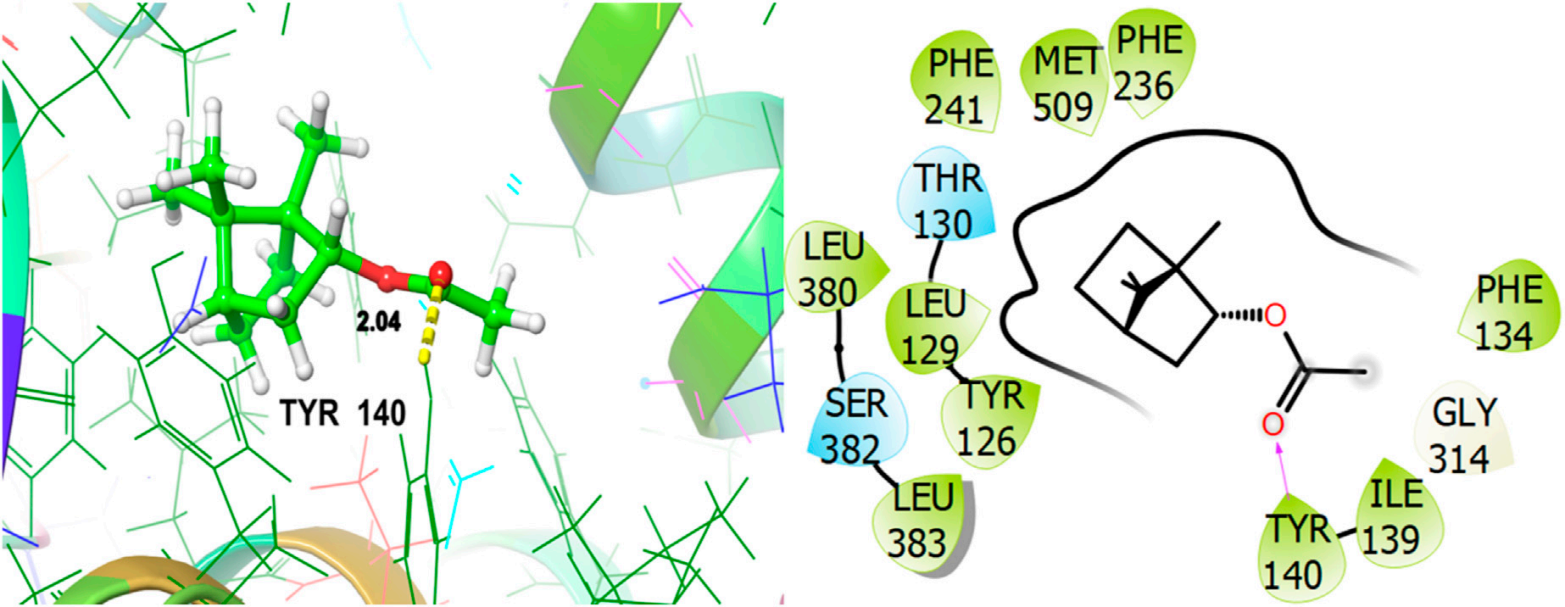
3D and 2D interactive view of Bornyl acetate (isolated from *T. articulate*) with antifungal target protein 5EQB.

To explore the antifungal activity, the co-crystallized ligand Figure 6, is showing a notable binding score as a GScore of-12.052 kcal/mol. It exhibits polar interactions with THR130, THR318, SER382, HIS468, THR507, SER508. HIS468 (2.24), CYS470 (2.09), and PHE506 (2.63) are involved in hydrogen bonding interactions at their relevant distances expressed in angstroms. Additionally, hydrophobic interactions were observed with ALA69, TYR72, LEU95, LEU96, TYR126, LEU129, PHE134, ILE139, TYR140, LEU147, VAL154, PHE236, PRO238, PHE241, LEU307, VAL311, LEU380, LEU383, PHE384, CYS470, ILE471, and MET509. The ligand named β-Eudesmol isolated from *J. phoenica* interacts with the antifungal target protein as is depicted in Figure 7. SER382 (1.86) is engaged in hydrogen bonding. Hydrophobic interactions are noted with TYR126, LEU129, PHE134, TYR140, PHE236, PRO238, PHE241, LEU380, LEU383, PHE384, and MET509. The polar interactions are shown by amino acid residues THR130, and SER382. The GScore is found to be −7.270 kcal/mol. Bornyl acetate isolated from *T. articulate* shows a GScore of −6.738 kcal/mol. TYR140 (2.04) is engaging in hydrogen bonding. The hydrophobic interactions are evident with TYR126, LEU129, PHE134, ILE139, TYR140, PHE236, PHE241, LEU380, LEU383, and MET509. The amino acids THR130, and SER382 show the polar contacts as shown in Figure 8.

**FIGURE 9:**
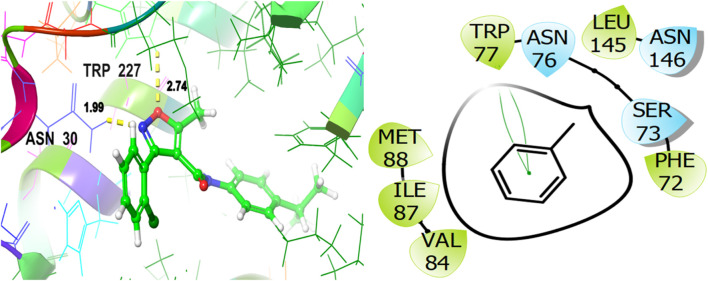
3D and 2D interactive view of co-crystallized ligand with antibacterial target protein 4Q9M.

**FIGURE 10:**
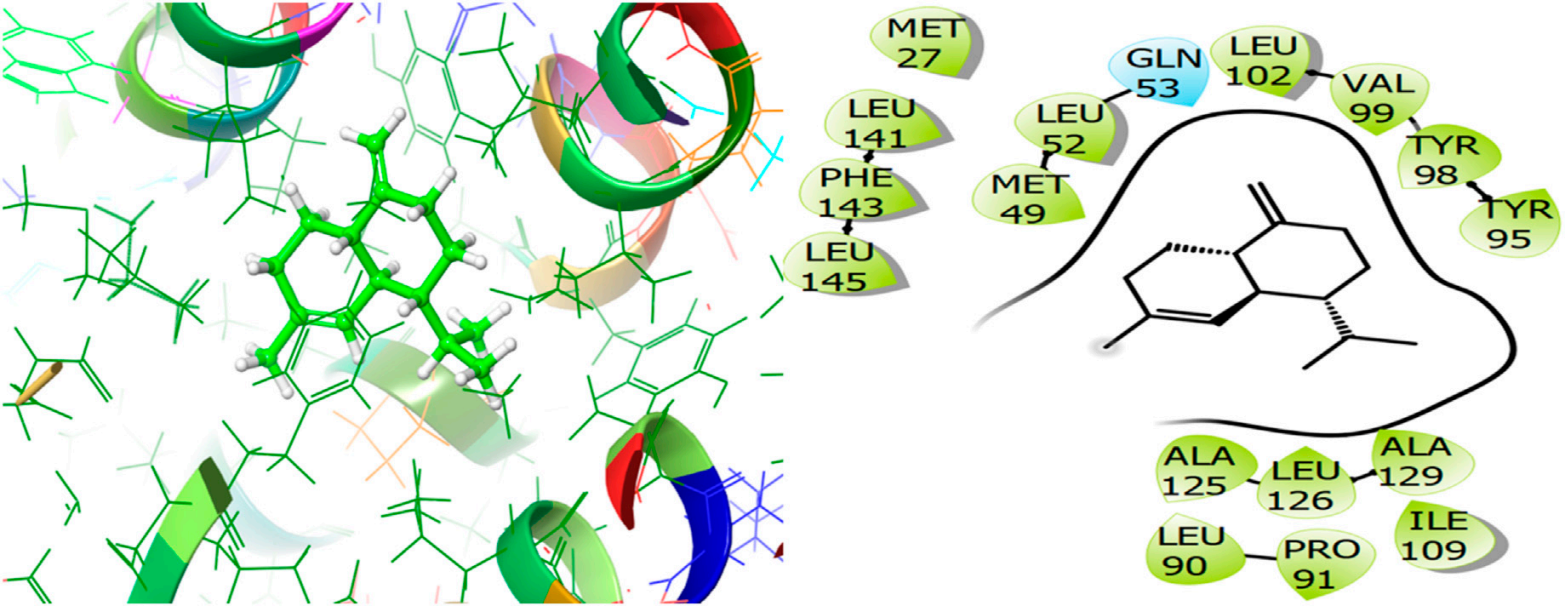
3D and 2D interactive view of γ- Cadinene (isolated from *J. phoenica*) with antibacterial target protein 4Q9M.

**FIGURE 11:**
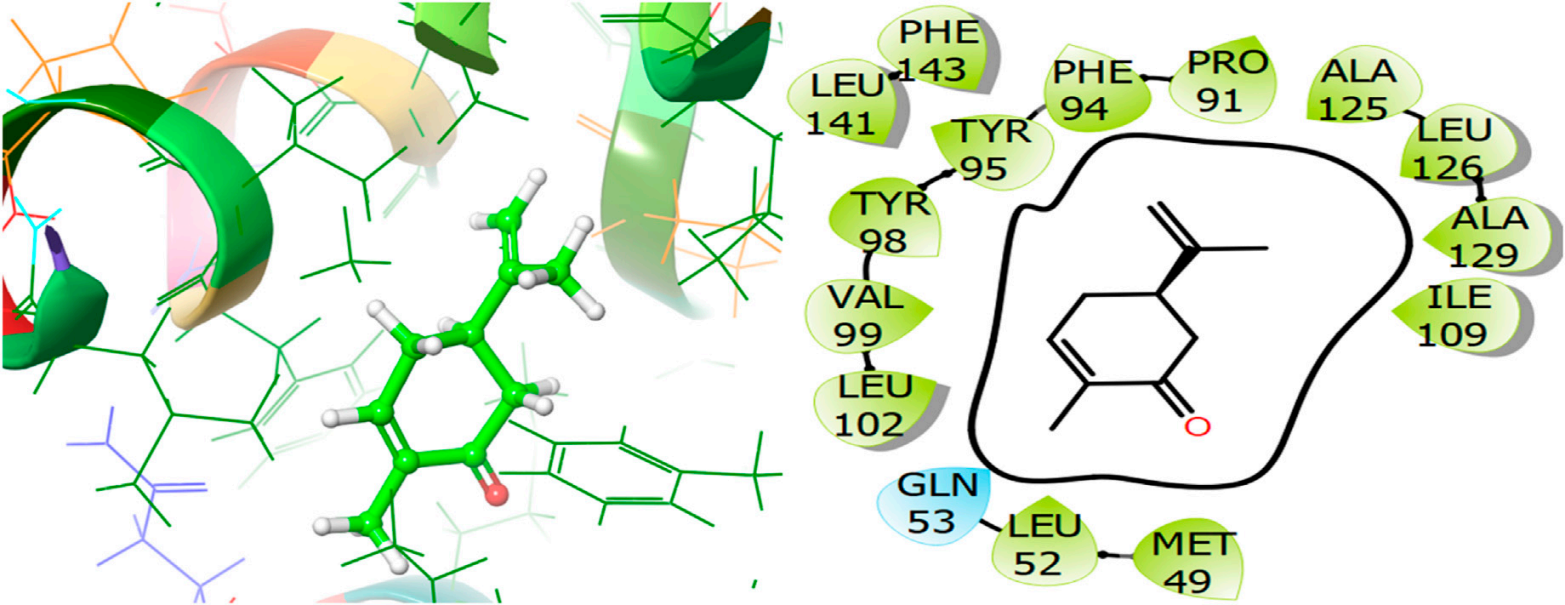
3D and 2D interactive view of Carvone (isolated from *T. articulate*) with antibacterial target protein 5EQB.

For the receptor 4Q9M (Antibacterial target protein) as shown in Figure 9, the co-crystallized ligand shows a binding score of GScore value of −7.597 kcal/mol. It exhibited polar interactions with SER73, ASN76, and ASN146 as well as forming hydrogen bonding interactions with amino acids ASN30 (1.99), and TRP227 (2.74). In addition, hydrophobic interactions are observed with PHE72, TRP77, VAL84, ILE87, MET88, and LEU145. The ligand γ- Cadinene isolated from *J. phoenica* interacts with the Q9M receptor is displayed in Figure 10. Hydrogen bonding is not observed. Hydrophobic interactions are observed with MET27, MET49, LEU52, LEU90, PRO91, TYR95, TYR98, VAL99, LEU102, ILE109, AL125, LEU126, ALA129, LEU141, PHE143, and LEU145. It shows polar interactions with GLN53. The GScore of α-Cadinene is found to be −8.63 kcal/mol. The carvone isolated from *T. articulate* shows a GScoreof −7.310 kcal/mol when docked with the antibacterial target protein. It is not involved in hydrogen bonding and GLN53 is responsible for polar contacts. The hydrophobic interactions are evident with MET49, LEU52, PRO91, PHE94, TYR95, TYR98, VAL99, LEU102, ILE109, AL125, LEU126, ALA129, LEU141, and PHE143 as shown in Figure 11.

**FIGURE 12:**
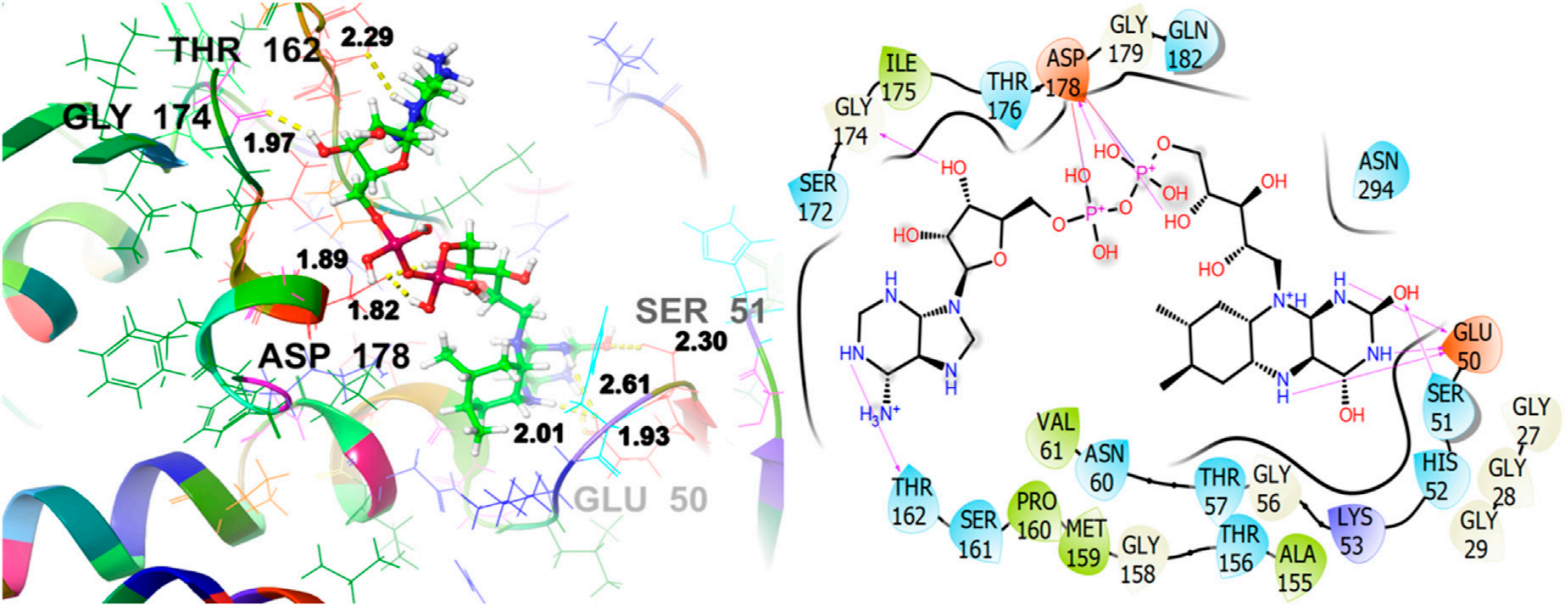
3D and 2D interactive view of co-crystallized ligand with antioxidant target protein1K4Q.

To reveal the antioxidant activity, the co-crystallized ligand of the target protein (1K4Q) Figure 12, shows a GScore of −8.628 kcal/mol. It exhibits polar interactions with SER51, HIS52, THR57, ASN60, THR156, SER161, THR162, SER172, THR176, GLN182, ASN294. GLU50 (1.93, 2.01, 2.61), SER51 (2.30), THR162 (2.29), GLY174 (1.97), and ASP178 (1.82, 1.89) are involved in hydrogen bonding interactions. The hydrophobic interactions are observed with VAL61, ALA155, MET159, PRO160, and ILE175.

**FIGURE 13:**
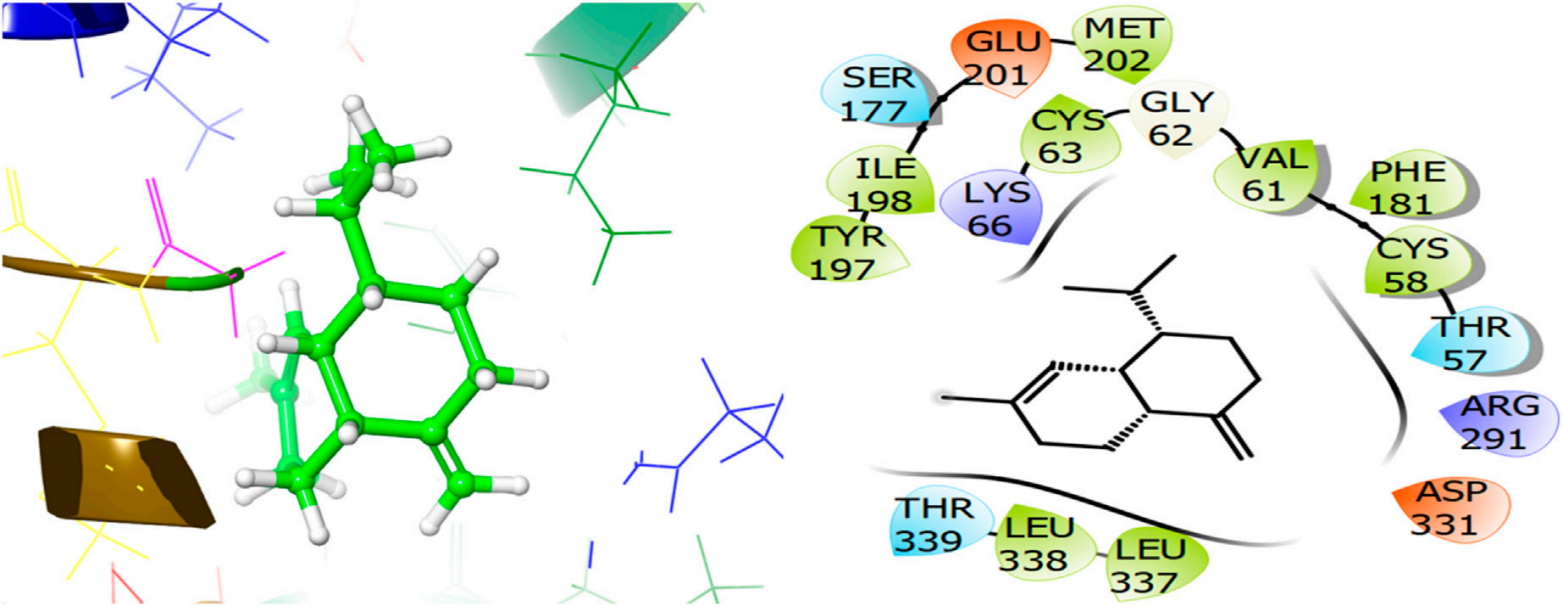
3D and 2D interactive view of γ-amorphene (isolated from *J. phoenica*) with antioxidant target protein 1K4Q.

**FIGURE 14:**
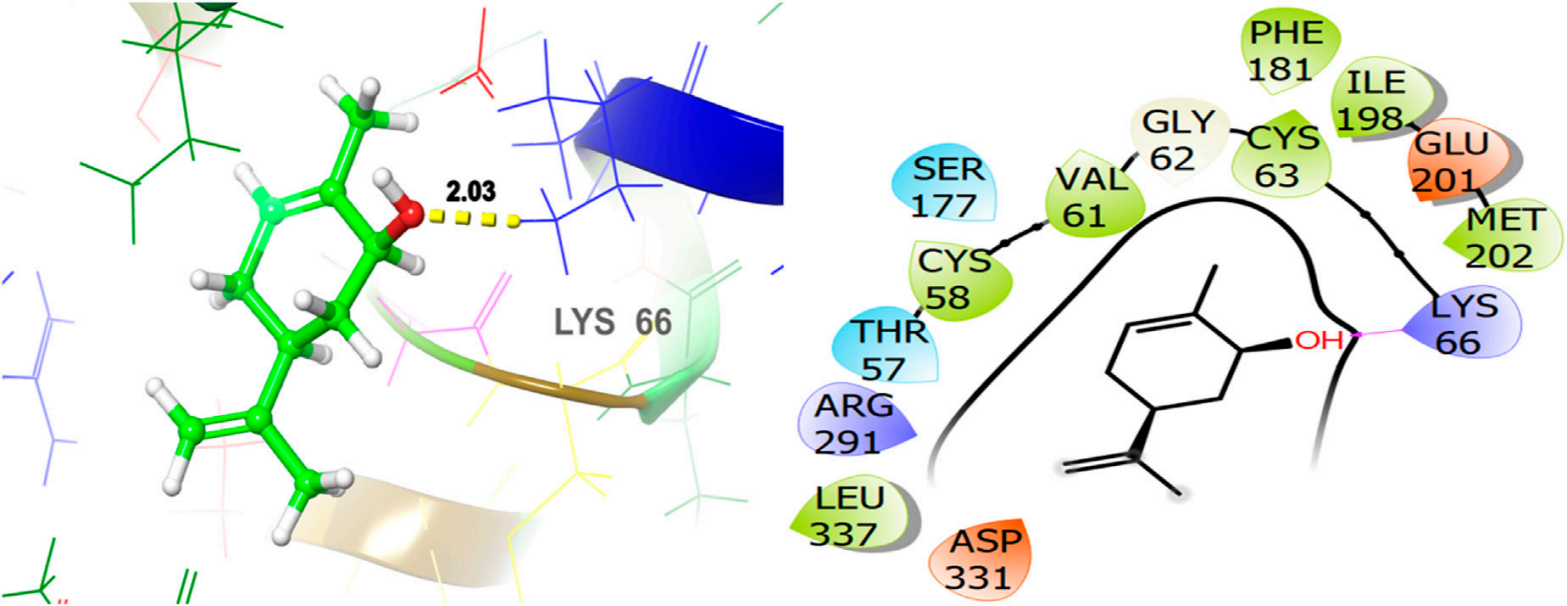
3D and 2D interactive view of Carveol <cis-> (isolated from *T. articulate*) with antioxidant target protein 1K4Q.

The ligand named γ-amorphene isolated from *J. phoenica* interacts with the antioxidant target protein as depicted in Figure 13. Hydrogen bonding is not evident. Hydrophobic interactions are noted with CYS58, VAL61, CYS63, PHE181, TYR197, ILE198, MET202, LEU337, and LEU338. The polar interactions are shown by amino acid residues THR57, SER177, and THR339. The GScore is found to be −5.346 kcal/mol. Carveol <cis-> isolated from *T. articulate* shows a GScore of −5.762 kcal/mol. LYS66 (2.03) is involved in hydrogen bonding. The hydrophobic interactions are evident with CYS58, VAL61, CYS63, PHE181, ILE198, MET202, and LEU337. The amino acids THR57, and SER177 show the polar contacts as shown in Figure 14.

## 4 Conclusion

For this study, we delved into the chemical composition and bioactivity of essential oils from *J. phoenica* and *T. articulata*, which are naturally found in the Middle Atlas region of Morocco. Our research suggests that these essential oils are primarily made up of terpenes, which could potentially explain their antioxidant and antimicrobial effects. The DPPH and FRAP assays uncovered significant reducing and anti-radical activities, indicating promising therapeutic applications. The experimental results were further supported by molecular docking simulations, which revealed potential interactions between the identified constituents and biological targets. Although this study offers valuable insights into the bioactivity of these essential oils, it is important to note that there are limitations. These include the use of only two plant species and a limited number of microbial strains. In comparison, the antibacterial and antioxidant characteristics of *T. articulata* and *J. phoenicea* essential oils are comparable, although their chemical makeup and bioactivity profiles differ significantly. *T. articulata* oil has a greater ability to scavenge free radicals and is more selective towards fungal strains than *J. phoenicea* oil, even though both oils exhibit remarkable potency against oxidative stress and microbial strains. *T. articulata* oil is dominated by sesquiterpenes, while *J. phoenicea* oil is rich in monoterpenes. These differences in the oils’ chemical profiles reflect their distinct phytochemical fingerprints and possible uses. Future research should broaden its focus to encompass a wider range of plant species, microbial strains, and *in vivo* studies to gain a comprehensive understanding of the therapeutic possibilities offered by these essential oils. However, our findings indicate that the essential oils of *J. phoenica* and *T. articulata* show potential as natural antimicrobial and antioxidant agents, especially the former. Further research is warranted to explore their capabilities.

## Data Availability

The raw data supporting the conclusions of this article will be made available by the authors, without undue reservation.

## References

[B1] Abbas Y. Ducousso M. Mohamed A. Azcón R. Duponnois R. (2006). Diversity of arbuscular mycorrhizal fungi in Tetraclinis articulata (vahl) masters woodlands in Morocco. Ann. For. Sci. 63 (3), 285–291. 10.1051/forest:2006007

[B2] Adams R. Bartel J. Price R. (2009). A new genus, Hesperocyparis, for the cypresses of the western hemisphere (CUPRESSACEAE). Phytologia 91.

[B3] Adams R. Pandey R. Rezzi S. Joseph C. (2002). Geographic variation in the Random Amplified Polymorphic DNAs (RAPDs) of Juniperus *phoenicea*, J.p. var. canariensis, J.p. subsp. eu-mediterranea, and J.p. var. turbinata. Biochem. Syst. Ecol. 30 (mars), 223–229. 10.1016/S0305-1978(01)00083-7

[B4] Adams R. P. (1998). The leaf essential oils and chemotaxonomy of Juniperus sect. Juniperus. Biochem. Syst. Ecol. 6 (26), 637–645. 10.1016/s0305-1978(98)00020-9

[B5] Adams R. P. (2000). Systematics of Juniperus section Juniperus based on leaf essential oils and random amplified polymorphic DNAs (RAPDs). Biochem. Syst. Ecol. 28 (6), 515–528. 10.1016/s0305-1978(99)00089-7 10793252

[B6] Adams R. P. Barrero A. F. Lara A. (1996). Comparisons of the leaf essential oils of*Juniperus phoenicea, J. phoenicea* subsp.*eu-mediterranea*Lebr. and thiv. and*J. phoenicea* var.*turbinata*(Guss.) parl. J. phoenicea subsp. eu-mediterranea Lebr. and Thiv. J. phoenicea var. turbinata (Guss.) Parl. J. Essent. Oil Res. 8 (4), 367–371. 10.1080/10412905.1996.9700642

[B7] Ait-Sidi-Brahim M. Markouk M. Larhsini M. (2019). “Moroccan medicinal plants as antiinfective and antioxidant agents,” in New look to phytomedicine (Elsevier), 91–142.

[B8] Ali H. Usman H. Ashraf W. Alqahtani F. Javaid S. Siddique F. (2023). Demaghi, a polyherbal formulation, mitigates aluminum chloride-induced neurological impairment in mice: insights from phytochemical analysis and behavioral assessment. Heliyon 9 (11), e21234. 10.1016/j.heliyon.2023.e21234 38027790 PMC10643107

[B9] Amalich S. Zekri N. Soro D. Fadili K. Khabbal Y. Mahjoubi M. (2015). Chemical characterization and antibacterial evaluation of Juniperus *phoenicea* L. Leaves and fruits’ essential oils from eastern high Atlas (Morocco). 13 881–889.

[B10] Amarti F. Satrani B. Aafi A. Ghanmi M. Farah A. Aberchane M. (2008). Composition chimique et activité antimicrobienne des huiles essentielles de Thymus capitatus et de Thymus bleicherianus du Maroc. Phytothérapie 6 (6), 342–347. 10.1007/s10298-008-0346-7

[B11] Amel B. (2013). Traditional treatment of high blood pressure and diabetes in Souk Ahras District. J. Pharmacogn. Phytotherapy 5 (1). 10.5897/JPP11.065

[B12] Angioni A. Barra A. Russo M. T. Coroneo V. Dessí S. Paolo C. (2003). Chemical composition of the essential oils of Juniperus from ripe and unripe berries and leaves and their antimicrobial activity. J. Agric. Food Chem. 51 (10), 3073–3078. 10.1021/jf026203j 12720394

[B13] Association Francaise de normalisation (1984). Recueil de normes Françaises des corps gras graines olágineuses, produits dérivés 3ème éd. Paris: AFNOR.

[B14] Atailia I. Djahoudi A. (2015). Composition chimique et activité antibactérienne de l’huile essentielle de géranium rosat (Pelargonium graveolens L’Hér.) cultivé en Algérie. Phytothérapie 13 (3), 156–162. 10.1007/s10298-015-0950-2

[B15] Azeem M. Hanif M. Mahmood K. Siddique F. Hashem H. E. Aziz M. (2023). Design, synthesis, spectroscopic characterization, *in-vitro* antibacterial evaluation and in-silico analysis of polycaprolactone containing chitosan-quercetin microspheres. J. Biomol. Struct. Dyn. 41 (15), 7084–7103. 10.1080/07391102.2022.2119602 36069131

[B16] Bekhechi C. Bekkara F. A. Consiglio D. Bighelli A. Tomi F. (2012). Chemical variability of the essential oil of Juniperus p*hoenicea* var. Turbinata from Algeria. Chem. and Biodivers. 9 (12), 2742–2753. 10.1002/cbdv.201200028 23255444

[B17] Bellakhdar J. Claisse R. Fleurentin J. Chafique Y. (1991). Repertory of standard herbal drugs in the Moroccan pharmacopoea. J. Ethnopharmacol. 35 (2), 123–143. 10.1016/0378-8741(91)90064-K 1809818

[B18] Benabid A. (2000). Flore et écosystèmes du Maroc: Evaluation et préservation de la biodiversité.

[B19] Berman H. M. Westbrook J. Feng Z. Gilliland G. Bhat T. N. Weissig H. (2000). The protein data bank. Nucleic acids Res. 28 (1), 235–242. 10.1093/nar/28.1.235 10592235 PMC102472

[B20] Boudjelal A. Henchiri C. Sari M. Sarri D. Hendel N. Benkhaled A. (2013). Herbalists and wild medicinal plants in M’sila (north Algeria): an ethnopharmacology survey. J. Ethnopharmacol. 148 (2), 395–402. 10.1016/j.jep.2013.03.082 23643544

[B21] Bourkhiss M. Chaouch A. Ouhssine M. Bourkhiss B. (2015). Étude physicochimique de l’huile essentielle de tetraclinis articulata (vahl) masters du plateau central marocain. Les Technol. Lab. 9:7. 10.34874/PRSM.teclab-vol9iss37.2823

[B22] Boussaid M. (2017). Caractérisation des huiles essentielles de Tetraclinis articulata (Vahl) Masters (Thuya de Barbarie) de la région de Tlemcen et étude de leurs activités biologiques. Thesis. 113.

[B23] Bouyahya A. Bakri Y. Khay E. Edaoudi F. Talbaoui A. Et-Touys A. (2017). Antibacterial, antioxidant and antitumor properties of Moroccan medicinal plants: a review. Asian Pac J. Trop. Dis. 7 (1), 57–64. 10.12980/apjtd.7.2017d6-294

[B24] Bouzouita N. Kachouri F. Ben Halima M. Chaabouni M. (2008). Composition chimique et activités antioxydante, antimicrobienne et insecticide de l’huile essentielle de *Juniperus phoenicea* . J. de Société Chimique de Tunisie 10 (janvier), 119–125.

[B25] Chikhoune A. Mohamed H. Kerbouche L. Baaliouamer A. Aissat K. (2013). Tetraclinis articulata (Vahl) Masters essential oils: chemical composition and biological activities. J. Essent. Oil Res. 25 (août), 300–307. 10.1080/10412905.2013.774625

[B26] Clevenger J. F. (1928). Apparatus for the determination of volatile oil. J. Am. Pharm. Assoc. 17, 345–349. 10.1002/jps.3080170407

[B27] Djahafi A. Taïbi K. Abderrahim L. A. (2021). Aromatic and medicinal plants used in traditional medicine in the region of Tiaret, North West of Algeria. Medit. Bot. 42, e71465. 10.5209/mbot.71465

[B28] Djouahri A. Boualem S. Boudarene L. Baaliouamer A. (2015). Geographic’s variation impact on chemical composition, antioxidant and anti-inflammatory activities of essential oils from wood and leaves of Tetraclinis articulata (vahl) masters. Industrial Crops Prod. 63 (janvier), 138–146. 10.1016/j.indcrop.2014.10.018

[B29] El M. Ahmed T. Gmouh S. Aberchane M. Benjouad A. Bakri Y. (2010). Chemical composition and bactericidal evaluation of essential oil of Tetraclinis articulata burl wood from Morocco. J. Indian Acad. Wood Sci. 7 (1-2), 14–18. 10.1007/s13196-010-0003-2

[B62] El Jemli M. Kamal R. Marmouzi I. Zerrouki A. Cherrah Y. Alaoui K. (2016). Radical-Scavenging Activity and Ferric Reducing Ability of *Juniperus Thurifera* (L.), *J. Oxycedrus* (L.), *J. Phoenicea* (L.) and *Tetraclinis Articulata* (L.). Adv. Pharmacol. Sci. 2016, 1–6. 10.1155/2016/6392656 PMC4884791 27293428

[B30] El-Sawi S. A. Motawae H. M. El-Sawi S. A. (2007). Chemical composition, cytotoxic activity and antimicrobial activity of essential oils of leaves and berries of Juniperus *phoenicea* L. grown in Egypt. Afr. J. Tradit. Complement. Altern. Med. 4 (4), 417–426. 10.4314/ajtcam.v4i4.31236 20161910 PMC2816504

[B31] Fahim M. Shrivastava B. Shrivastava A. Ibrahim M. Parveen R. Ahmad S. (2017). Review on extraction methods, antioxidant and antimicrobial properties of volatile oils. Ann. Phytomed 6 (2), 5–46. 10.21276/ap.2017.6.2.2

[B32] Farjon A. (2005). A monograph of Cupressaceae and sciadopitys. Kew: Royal Botanic Gardens.

[B33] Farjon A. (2010). A handbook of the world’s conifers. Available at: https://www.worldcat.org/fr/title/handbook-of-the-worlds-conifers/oclc/833766061.

[B34] Fennane M. (1957). Flore pratique du Maroc: manuel de détermination des plantes vasculaires. Institut scientifique.

[B35] Friesner R. A. Murphy R. B. Repasky M. P. Frye L. L. Greenwood J. R. Halgren T. A. (2006). Extra precision glide: docking and scoring incorporating a model of hydrophobic enclosure for protein− ligand complexes. J. Med. Chem. 49 (21), 6177–6196. 10.1021/jm051256o 17034125

[B36] Ghanmi M. Satrani B. Chaouch A. Aafi A. El Abid A. Rchid Ismaili M. (2007). Composition Chimique et Activité Antimicrobienne de l’essence de Térébenthine Du Pin Maritime (Pinus Pinaster) et Du Pin d’Alep (Pinus Hale- Pensis) Du Maroc. Acta Bot. Gallica 154 (2), 293–300. 10.1080/12538078.2007.10516058

[B37] Ghnaya B. Amri I. Hanana M. Gargouri S. Jamoussi B. Romane A. (2016). Tetraclinis articulata (vahl.) masters essential oil from Tunisia: chemical characterization and herbicidal and antifungal activities assessment. Industrial Crops Prod. 83 (mai), 113–117. 10.1016/j.indcrop.2015.12.026

[B39] Harmouzi A. Ahmed B. El Ammari Y. Abdelaziz C. (2016). Chemical composition and toxicity of Moroccan Tetraclinis articulata and Juniperus p*hoenicea* essential oils against Aphis citricola goot, 1912 (Homoptera, aphididae). Res. Chem. Intermed. 42 (9), 7185–7197. 10.1007/s11164-016-2528-5

[B40] Karagözler A. A. Erdag B. Emek Y. Uygun D. (2008). Antioxidant activity and proline content of leaf extracts from dorystoechas hastata. Food Chem. 111 (2), 400–407. 10.1016/j.foodchem.2008.03.089 26047442

[B41] Lawal O. A. Ogunwande I. A. (2013). “Essential oils from the medicinal plants of Africa,” in Medicinal plant research in Africa (Elsevier), 203–224.

[B42] Lu C. Wu C. Ghoreishi D. Chen W. Wang L. Damm W. (2021). OPLS4: improving force field accuracy on challenging regimes of chemical space. J. Chem. theory Comput. 17 (7), 4291–4300. 10.1021/acs.jctc.1c00302 34096718

[B43] Luo B. Li D. Zhang A.-L. Gao J.-M. (2018). Synthesis, antifungal activities and molecular docking studies of benzoxazole and benzothiazole derivatives. Molecules 23 (10), 2457. 10.3390/molecules23102457 30257495 PMC6222379

[B44] Mansouri N. Satrani B. Mohamed G. El Ghadraoui L. (2011). Etude chimique et biologique des huiles essentielles de Juniperus *phoenicea* ssp. lycia et Juniperus *phoenicea* ssp. turbinata du Maroc. Biotechnol. Agron. Soc. Environ. 10.

[B46] Moldão-Martins M. Bernardo-Gil G. M. Da Costa L. M. (2002). Sensory and chemical evaluation of thymus zygis L. Essential oil and compressed CO2 extracts. Eur. Food Res. Technol. 214 (3), 207–211. 10.1007/s00217-001-0451-4

[B47] Nasir Shah S. Khan I. Tul Muntaha S. Hayat A. Ur Rehman M. Ali Shah T. (2023). Bioactive, antioxidant and antimicrobial properties of chemically fingerprinted essential oil s extracted from Eucalyptus globulus: *in-vitro* and in-silico investigations. Front. Chem. 11, 1287317. 10.3389/fchem.2023.1287317 38188929 PMC10768562

[B63] Ouis N. (2015). Etude Chimique et Biologique des Huiles Essentielles de coriandre, de fenouil et de persil. Thèse de Doctorat. Université d’Oran 1, 37.

[B48] Parejo I. Codina C. Petrakis C. (2000). Evaluation of scavenging activity assessed by Co(II)/EDTA-Induced luminol chemiluminescence and dpphá (2,2-diphenyl-1-picrylhydrazyl) free radical assay. J. Pharmacol. Toxicol. Methods 6. 10.1016/S1056-8719(01)00110-1 11395328

[B49] Rabib H. Elagdi C. Hsaine M. Fougrach H. Koussa T. Wadi B. (2020). Antioxidant and antibacterial activities of the essential oil of Moroccan Tetraclinis articulata (vahl) masters. Biochem. Res. Int. 2020, 1–6. 10.1155/2020/9638548 PMC7360168 32704398

[B50] Rice-Evans C. A. Miller N. J. Bolwell P. G. Bramley P. M. Pridham J. B. (1995). The relative antioxidant activities of plant-derived polyphenolic flavonoids. Free Radic. Res. 22 (4), 375–383. 10.3109/10715769509145649 7633567

[B51] Saber M. Menyiy N. E. Charfi S. Mrabti H. N. Belmehdi O. El Moudden H. (2023). Comprehensive overview on nutritional, phytochemistry and pharmacological properties of Tetraclinis articulata Masters. Food Rev. Int. 39 (7), 3691–3752. 10.1080/87559129.2021.2013257

[B52] Sadiki F. Z. El Idrissi M. Sbiti M. Lemrhari A. Trifan A. Cioanca O. (2018). Chemical composition and antibacterial activity of essential oil of Tetraclinis articulata (vahl) masters branches of eastern Morocco. Chem. Biol. Technol. Agric. 5 (1), 24. 10.1186/s40538-018-0137-9

[B53] Sahib N. Boumediene M. Abid M. Mihamou A. Serghini-Caid H. Elamrani A. (2022). Phenotypic comparison of three populations of Juniperus turbinata guss. in North-eastern Morocco. Forests 13 (2), 287. 10.3390/f13020287

[B54] Sanchez-Moreno C. (2002). Review: methods used to evaluate the free radical scavenging activity in foods and biological systems. Food Sci. Technol. Int. 8 (juin), 121–137. 10.1177/1082013202008003770

[B55] Satrani B. Ghanmi M. Farah A. Aafi A. Fougrach H. Bourkhiss B. (2007). Composition chimique et activité antimicrobienne de l’huile essentielle de cladanthus mixtus. Available at: https://www.semanticscholar.org/paper/COMPOSITION-CHIMIQUE-ET-ACTIVIT%C3%89-ANTIMICROBIENNE-DE-Satrani-Ghanmi/d04642ea61bc7431048621b7383d5446fe56ed1b.

[B56] Tahar K. Zakia K. Nadia T. Omar A. (2023). Insecticidal activity of Tetraclinis articulata L. essential oils on *Tribolium castaneum* (Herbst 1797)(Tenebrionidae: Coleoptera) adults. Indian J. Ecol. 50 (2), 462–467. 10.1017/S1742758411000221

[B57] Tepe B. Sokmen M. Askin Akpulat H. Daferera D. Polissiou M. Atalay S. (2005). Antioxidative activity of the essential oils of thymus sipyleus subsp. sipyleus var. Sipyleus and thymus sipyleus subsp. sipyleus var. Rosulans. J. Food Eng. 66 (4), 447–454. 10.1016/j.jfoodeng.2004.04.015

[B58] Thibaut Joliet (2015). Séchage des plantes aromatiques et médicinales.

[B59] Yusuf A. J. Adegboyega A. E. Yakubu A. H. Johnson G. I. Asomadu R. O. Adeduro M. N. (2023). Exploring Scutellaria baicalensis bioactives as EGFR tyrosine kinase inhibitors: cheminformatics and molecular docking studies. Inf. Med. Unlocked 43, 101406. 10.1016/j.imu.2023.101406

[B60] Zerkani H. Tagnaout I. Dirioiche A. Adadi I. El Karkouri J. Padzys G. S. (2019). Chemical characterization and antibacterial activity of the essential oils of Tetraclinis articulata (vahl) from Morocco. Mediterr. J. Chem. 8 (5), 390–396. 10.13171/mjc851907076hz

[B61] Zheng M. Liu X. Xu Y. Li H. Luo C. Jiang H. (2013). Computational methods for drug design and discovery: focus on China. Trends Pharmacol. Sci. 34 (10), 549–559. 10.1016/j.tips.2013.08.004 24035675 PMC7126378

